# Risk Factors for Recurrence, Complications and Mortality in *Clostridium difficile* Infection: A Systematic Review

**DOI:** 10.1371/journal.pone.0098400

**Published:** 2014-06-04

**Authors:** Claire Nour Abou Chakra, Jacques Pepin, Stephanie Sirard, Louis Valiquette

**Affiliations:** Department of Microbiology and Infectious Diseases, Université de Sherbrooke, Sherbrooke, Quebec, Canada; Universidad Andres Bello, Chile

## Abstract

**Background:**

*Clostridium difficile* infection (CDI) can lead to complications, recurrence, and death. Numerous studies have assessed risk factors for these unfavourable outcomes, but systematic reviews or meta-analyses published so far were limited in scope or in quality.

**Methods:**

A systematic review was completed according to PRISMA guidelines. An electronic search in five databases was performed. Studies published until October 2013 were included if risk factors for at least one CDI outcome were assessed with multivariate analyses.

**Results:**

68 studies were included: 24 assessed risk factors for recurrence, 18 for complicated CDI, 8 for treatment failure, and 30 for mortality. Most studies accounted for mortality in the definition of complicated CDI. Important variables were inconsistently reported, such as previous episodes and use of antibiotics. Substantial heterogeneity and methodological limitations were noted, mainly in the sample size, the definition of the outcomes and periods of follow-up, precluding a meta-analysis. Older age, use of antibiotics after diagnosis, use of proton pump inhibitors, and strain type were the most frequent risk factors for recurrence. Older age, leucocytosis, renal failure and co-morbidities were frequent risk factors for complicated CDI. When considered alone, mortality was associated with age, co-morbidities, hypo-albuminemia, leucocytosis, acute renal failure, and infection with ribotype 027.

**Conclusion:**

Laboratory parameters currently used in European and American guidelines to define patients at risk of a complicated CDI are adequate. Strategies for the management of CDI should be tailored according to the age of the patient, biological markers of severity, and underlying co-morbidities.

## Introduction

Highly associated with exposure to antibiotics, *Clostridium difficile* infection (CDI) causes 20 to 30% of antibiotic-associated diarrhea and is the most common cause of nosocomial diarrhoea [1]–[4]. The risk of CDI increases up to 6-fold during antibiotic therapy and in the subsequent month [5], [6]. In the early 2000s, a renewed interest in CDI followed the emergence of a hypervirulent strain (NAP1/BI/027) associated with frequent recurrences and higher severity [7], [8]. Several novel treatments of CDI are being studied, some of which have been associated with a lower risk of recurrence [9]–[11].

Identifying clinical parameters or host-related factors associated with adverse outcomes would improve the management of CDI in the early stage of the disease. In a previous systematic review [12], we showed that several studies used empirically-defined risk factors for the derivation of clinical prediction rules for unfavourable outcomes of CDI, while others used univariate comparisons between CDI and non-CDI groups. Few clinical variables remained significant in multivariate analyses.

Risk factors for unfavourable outcomes of CDI have been studied before and after the emergence of NAP1/BI/027. To our knowledge, only one systematic review with a meta-analysis, published in 2008, has addressed risk factors for recurrence with a search limited to PubMed [13]. More recently, a systematic review of risk factors for mortality pooled results of univariate and multivariate analyses of hospital-based studies [14]. Two other reviews that ascertained CDI-related mortality were performed but specific risk factors were not reported [15], [16]. Consequently, we performed a systematic review of all publications that identified risk factors for recurrence, treatment failure, complications and/or mortality in patients diagnosed with CDI.

## Methods

### Search strategy and selection criteria

A systematic review was performed according to PRISMA guidelines [17] (Checklist S1) using an electronic search of all studies published from January 1978 until October 2013. The search was limited to human studies and used the following online libraries and databases: MEDLINE, PubMed, Cochrane Library for evidence based-medicine, Embase and Web of Science (Text S1). The final electronic search was performed on 21 October 2013. Publications from all sources were merged into one file and duplicates were removed. A first screening of titles and abstracts followed by a full-text review were performed. In addition, the reference lists of identified studies were searched manually.

We included studies that: i) targeted *C. difficile* as the main pathogen; ii) measured at least one relevant outcome: severity, complications, mortality, treatment failure and/or recurrence; iii) identified risk factors for the main outcome(s) using risk assessment measures such as odds ratios (OR), relative risks or ratios (RR) and hazard ratios (HR). Any complication, fulminant colitis, ICU admission, shock, and/or death (when used as part of a composite outcome) were grouped under “complicated CDI”. We excluded all studies that used only univariate comparisons of groups, aimed to develop a risk stratification tool or a predictive model [12], and those conducted exclusively in children, in populations with selected pathologies or undergoing particular procedures (e.g. organ transplants, CT-scans, or endoscopies).

### Data extraction

Two reviewers (CAC and SS) extracted the following data into a standardized matrix: year of publication, location, year of diagnosis, type of tests for the laboratory diagnosis of CDI, definition and frequency of the outcome(s) of interest, study design, duration of follow-up, population and comparison groups, sample size, statistical analyses, number of variables and number of events per variable (EPV) in the final model, and main results in relation with the objectives of the review. Correspondences requesting clarifications were sent to authors in case of missing or incomplete data (n = 9).

Studies that assessed two or more outcomes were allocated to each category of outcomes. Results from included studies were plotted using GraphPad Prism 6.01 (GraphPad Software, San Diego, CA). Due to the small number of studies assessing common risk factors for defined outcomes, ORs, RRs and HRs with their confidence intervals (CI) are reported in the same forest plots. Some factors such as multi-organ failure or other severe medical status immediately preceding mortality were considered too closely related to death in the pathogenic pathway and were therefore not considered as risk factors in this review.

### Risk of bias assessment

A quality control process was performed on 10% of the first screening of abstracts (LV), as well as on included studies. Reviewers had a good agreement concerning eligible studies and final inclusion (87%). Disagreements were resolved by a third party (JP).

Two methods were used for the assessment of the individual and overall risk of bias across studies: i) the number of EPV (recurrence, treatment failure, complications, and/or death) in the final multivariate model of each study, assuming that at least 10 EPV are necessary [18], [19]; ii) relevant clinical and epidemiological variables in relation with CDI in each study, and adjustment for these variables in multivariate analyses. The main variables were: confirmed diagnosis of CDI, age, gender, the site of acquisition of the infection (SI), co-morbidities, occurrence and number of previous episode(s) of CDI (PE), recent antibiotic therapy (AB), immunosuppression (IS), use of anti-ulcer medication (AU), recent surgery or procedure (RS), and blood tests (white cell count, haematocrit, serum lactate, serum albumin, serum creatinine, and C-reactive protein).

## Results

**Figure 1:**
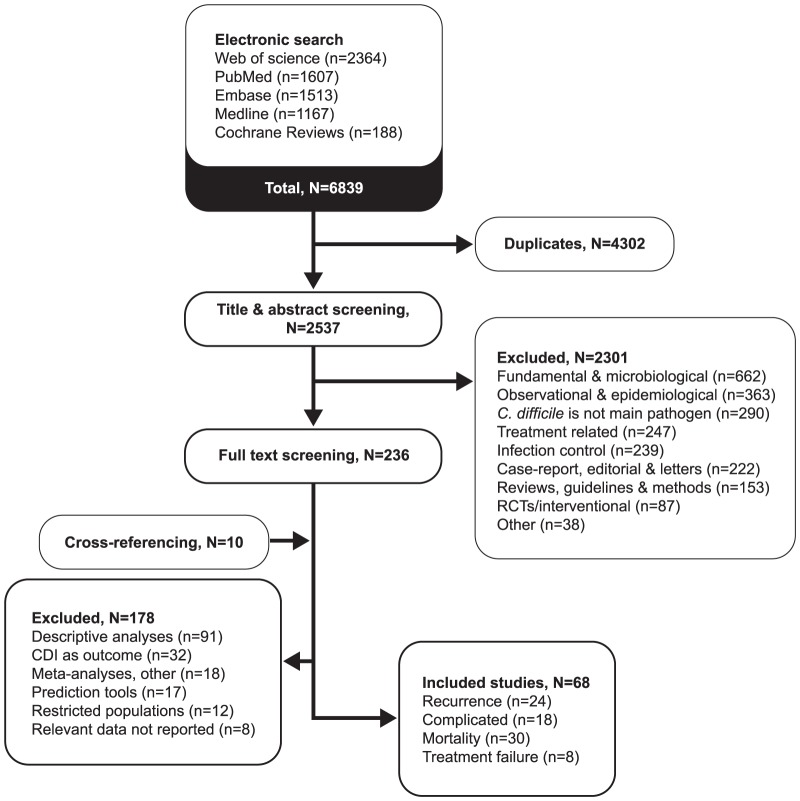
Flowchart of included and excluded publications.

The electronic search led to 6839 publications. After excluding duplicates, 2537 were reviewed by their title and abstract (Figure 1), among which 2301 were excluded at the first screening and 178 after full-text verification. We included in this review 68 studies that examined risk factors for one or more outcomes: 19 assessed risk factors for recurrence only, 11 for complicated CDI only (including or not mortality), two for treatment failure only, 23 for mortality alone (among them six in patients needed colectomy), and 13 for multiple outcomes (including six for treatment failure). The characteristics of included studies are shown in tables S1 to S4. The majority of included studies used retrospective cohorts (45; 66%), 15 used prospective cohorts (22%), four were retrospective case-control studies (6%), and four were clinical trials (6%). Except for six studies using administrative databases [20]–[26], sample sizes were small with a median of 128 patients (range 13-2042). Most studies (14/18) on complicated CDI included death (mostly all-cause 30-day mortality) within a composite outcome. The method used for CDI toxin detection was reported in 94% (n = 64) of studies: Toxin A and B enzyme immunoassay (EIA) was used in 39% (n = 25), direct cytotoxin assay (CTA) in 19% (n = 12), toxin A EIA in 9% (n = 6), polymerase chain reaction (PCR) in 9% (n = 6), toxigenic culture in 5% (n = 3), unspecified toxin assay in 11% (n = 7), and combined approaches in 12% (n = 8).

Overall, the risk of experiencing at least one recurrence ranged between 12% and 64% (median 22%; n = 26 studies). Risk of a complicated CDI (including or not death) ranged between 7% and 48% (median 18%; n = 15), and treatment failure between 5% and 50% (median 21%; n = 9). In studies on mortality alone, risk of 30-day mortality ranged between 8% and 53% (median 19%; n = 14). Predictably, mortality was higher in selected patients who needed an emergency colectomy (median 38%, range = 31–46%; n = 6) or ICU admission (median 36%, range = 28%–53%; n = 4).

### Analysis of risk factors

#### 1. Risk factors for recurrence (24 studies)

**Figure 2:**
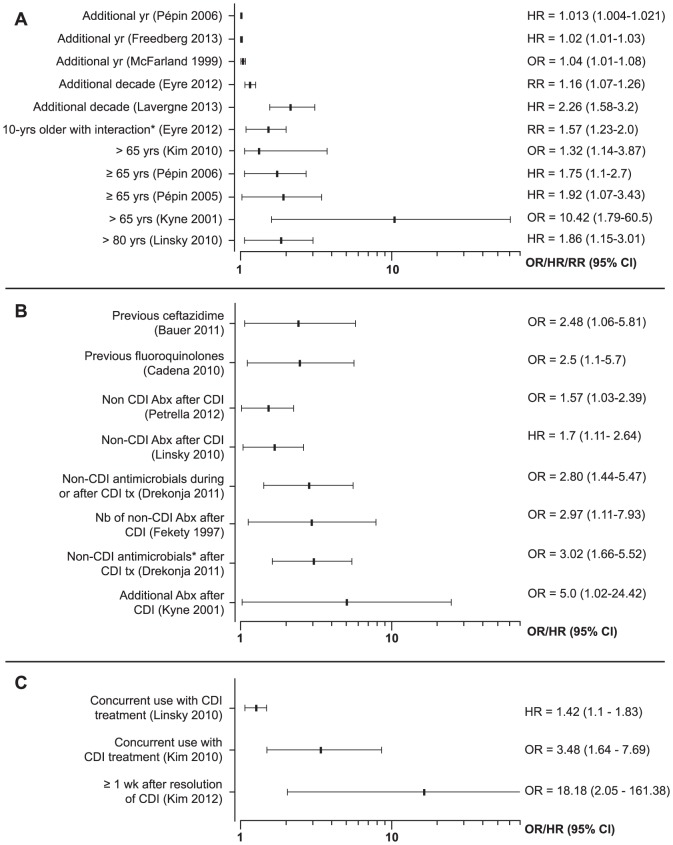
Forest plots of associations of age, antibiotic use and PPIs with recurrence of CDI. *Effect of age in deciles in interaction with previous dialysis/chemotherapy. *Non-CDI antimicrobial within 30-days of completing treatment for CDI.

Recurrent CDI was assessed through pre-defined follow-up performed at 60 and 90 days after diagnosis in only four prospective cohorts and four clinical trials (Table S1). The interval between the recurrent and the first episodes varied between 2 and 180 days after completion of therapy. Frequent risk factors for recurrence are shown in Figure 2: age (9 studies) (Figure 2A), antibiotics during or after CDI diagnosis (7 studies) (Figure 2B), and use of PPIs (3 studies) (Figure 2C). The relative risk for recurrence ranged between 1.01 and 1.04 for each additional year of age [26]–[29],_ENREF_27 between 1.3 and 10.4 with age >65 years [26], [30]–[32], between 1.6 and 5.0 with use of antibiotics after CDI [20], [32]–[34], and between 1.4 and 18.2 with use of PPIs [20], [30], [35]. In four studies with different typing methods [33], [36]–[38], the hypervirulent strain (NAP1/BI/027) was associated with recurrence (Table 1), but this association was not significant in a study using genome sequencing [25].

**Table 1:** Association between unfavourable outcomes and strain type in multivariate analyses.

Study	Typing method	Strain type	Period of data collection	N of strains	% of strain (n)	OR/HR/RR (95% CI)
Recurrence						
Petrella 2012 [33]/Louie 2013 [36]	REA	BI vs. non-BI	2007–2009	719	34 (247)*	1.57 (1.01–2.5)/1.6 (1.03–2.5)
Stewart 2013 [37]	PCR toxinotyping/ribotyping and tcdC genotyping	Tox A^+^B ^+^ CDT/Tox A^+^B ^+^ CDT + tcdC deletion	NR	69	61 (42)^**^/ 56 (39)^**^	3.1 (2.97–3.3)/5.3 (3.5–6.1)
Marsh 2012 [38]	MLVA and tcdC genotyping	tcdC-I genotype (ribotype 027) vs. other	2001–2009	82	45 (37)	6.9 (1.7–28.2)
Eyre 2012 [25]	MLST	Ribotype 027 vs. clade 1^a^	2006–2010	1076	28 (300)	1.2 (0.9–1.5)
Complicated CDI						
Bauer 2011 [46]	PCR toxinotyping and ribotyping		2008	389		
		Ribotype 018 vs. others			6 (23)	6.2 (1.28–29.8)
		Ribotype 056 vs. others			2 (6)	13.0 (1.1–148.3)
		Ribotype 015 vs. others			3 (13)	4.6 (0.98–21.2)
		Ribotype 027 vs. others			5 (19)	2.6 (0.6–10.2)
		Ribotype 014/020 vs. others			16 (61)	0.6 (0.2–2.2)
Søes 2012 [49]	PCR toxinotyping/ribotyping and tcdC genotyping	Tox A^+^B ^+^CDT^+^ vs. A^+^B^+^CDT^−^	2006–2007	82	26 (21)	6.0 (1.5–23.8)
Walk 2012 [48]	PCR toxinotyping/ribotyping	Ribotype 027/078 vs. others	2010–2011	310	14 (43)	0.8 (0.07–10.0)
Rao 2013 [47]	PCR toxinotyping/ribotyping	Ribotype 027 vs. others	2010–2012	22	32 (7)	2.7 (0.3–25.3)
30-day mortality						
Inns 2013 [21]	PCR	Ribotype 027 vs. infrequent ^b^	2009–2011	1426	10 (147)	1.3 (1.02–1.7)
Labbé 2008 [55]	PCR toxinotyping and ribotyping	Ribotype 027 vs. others	2000–01 & 2003–04 (outbreak)	230 175	61 (141) 29 (41)	2.1 (1.0–4.2)/7.5 (1.6–35.5)
Walker 2013 [23]	MLST, correlation with ribotypes	Ribotype 027 vs. clade 1^a^	2006–2011	1893	20 (560)	3.4 (2.5–4.7)
Huttunen 2012 [56]	PCR	Ribotype 027 vs. others^c^	2008–2010	780	14 (111)	4.6 (1.4–15)
Goorhuis 2011 [57]	MLVA and STRD	Ribotype 027 vs. others^d^	2005–2007	168	27 (46)	10.5 (1.2–92)
Inns 2013 [21]	PCR	Ribotype 015 vs. infrequent ^b^	2009–2011	1426	8 (111)	0.5 (0.3–0.8)
Goorhuis 2011 [57]	MLVA and STRD	Ribotype 017 vs. others^d^	2005–2007	168	34 (57)	8.9 (1.04–75.8)
Walker 2013 [23]	MLST, correlation with ribotypes	Ribotype 078 vs. clade 1^b^	2006–2011	1893	2 (63)	5.4 (3.1–9.3)
Søes 2012 [49]	PCR toxinotyping/ribotyping and tcdC genotyping	Tox A^+^B ^+^CDT^+^ vs. A^+^B^+^CDT^−^	2006–2007	82	26 (21)	1.0 (0.2–5.1)

REA = Restriction endonuclease analysis. PCR = Polymerase chain reaction. MLVA = Multiple-Locus Variable number tandem repeat Analysis. STRD = Summed Tandem-Repeat Difference. MLST =  Multilocus Sequence Typing. NR =  not reported;

^*^Overall % of strain BI in the cohort, the % in the sub-population used for multivariate analyses was not reported.^ **^ % of binary toxin gene and *tcd*C mutation respectively, the % of combinations were not reported.

a Comparison of clade 2 with 99% PCR ribotype 027 vs. clade 1, and clade 5 with 100% PCR ribotype 078 vs. clade 1.^ b^ Compared to infrequent ribotypes in the study (other than R01, 02, 05, 015, 016, 023, 027, 064, 078 and 106).^ c^ Hypervirulent strain vs. non-hypervirulent, ribotype 027 was prevailing during the study period.^ d^ Other ribotype: non-027 and non-017.

**Figure 3:**
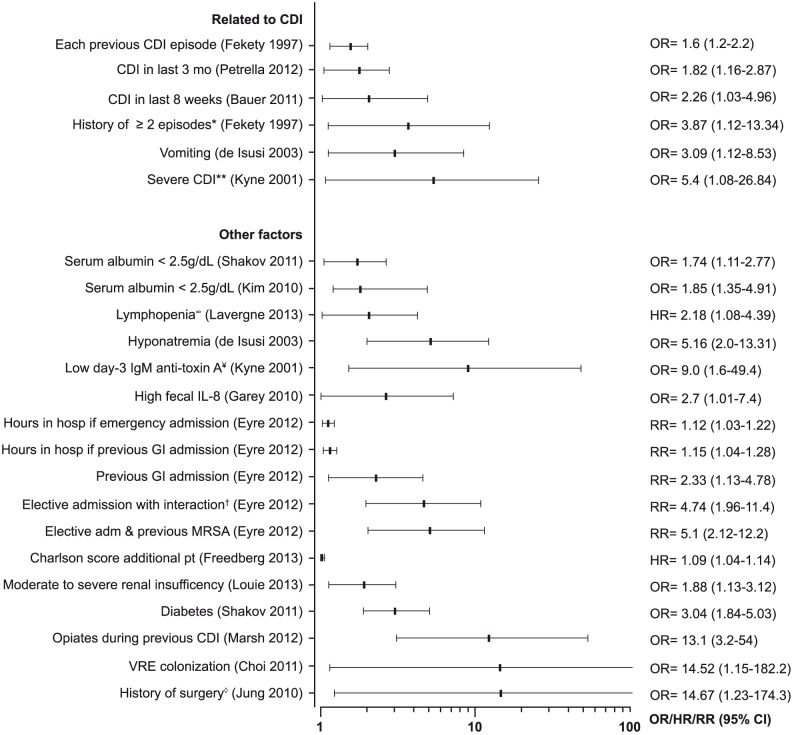
Other risk factors for recurrence of CDI. * History of recurrence vs. new CDI. ** Modified Horn’s index (3 pts). ∞ Lymphopenia at completion of CDI treatment: Absolute cell count <1.0×10^9^/L. ¥<2.22 ELISA units, adjusted on disease severity. † Elective admission vs. emergency if previous dialysis/chemotherapy (interaction). ◊ History of surgery within 1 month before CDI treatment. MRSA = previous methicillin-resistant *Staphylococcus aureus* (interaction). VRE = vancomycin-resistant enterococci.

Risk of recurrence was inconsistently associated with the site of acquisition: community-acquisition of CDI was highly associated with recurrence in one study (OR = 11.2; p = 0.02) [39],while acquiring CDI in hospital and each additional day of hospitalization were risk factors in two others (HR = 1.5; 95% CI = 1.1–2.1 and HR = 1.01; 95% CI = 1.0–1.02, respectively) [26], [31]. Many other risk factors were examined, and among them three were considered as related to recurrent CDI, but each in only one or two studies (Figure 3). The role of the immune response was addressed in only three studies (Figure 3) [29], [32], [40], but all showed that recurrence was associated with low antibody titres (IgM and IgG anti-toxin A, and IL-8) [32], [40], and a positive *C. difficile* antitoxin serology (HR = 0.17; 95% CI = 0.05–0.59) [29].

#### 2. Risk factors for complicated CDI (18 studies)

**Figure 4:**
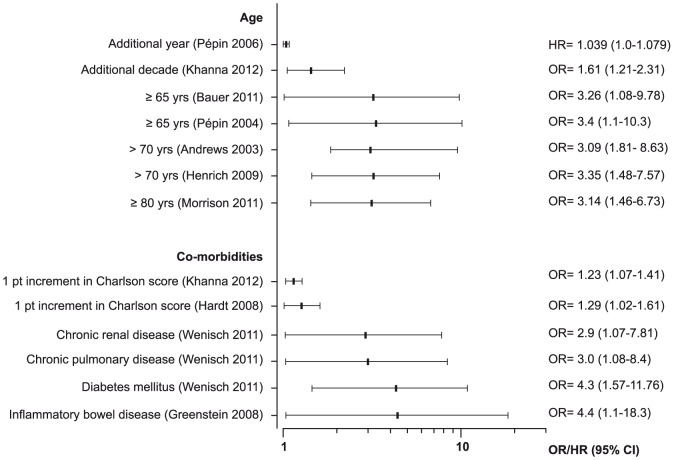
Forest plots of reported associations with complicated CDI: age and co-morbidities or health status.

**Figure 5:**
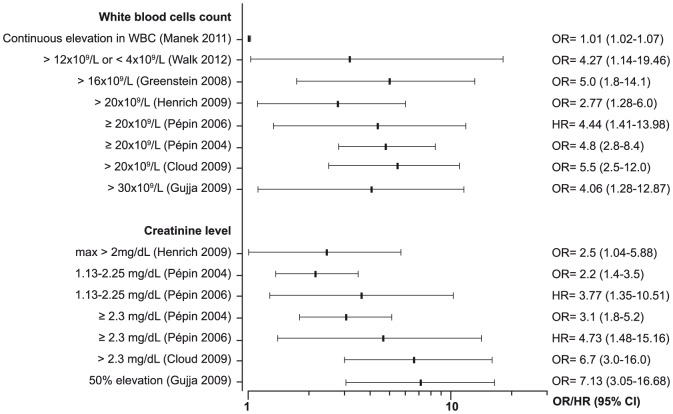
Forest plots of reported associations with complicated CDI: white blood cells count (WBC) and creatinine levels. WBC units were converted to the international system unit (10^9^/L). Creatinine levels were converted to the conventional unit using the formula: Creatinine [mg/dL]  =  creatinine/88.4 [µmol/L].

The definition of complicated CDI varied between studies, resulting in much heterogeneity (Table S2 and Table S3). Frequent risk factors identified in several studies were: older age and underlying co-morbidities (7 and 4 studies respectively) (Figure 4), high leucocyte count (8 studies) and acute renal failure (5 studies) (Figure 5). The relative risk of complicated CDI ranged between 2.7 and 5.5 with leucocytes count >20×10^9^/L [41]–[43], and between 3.1 and 6.7 with creatinine >2.3 mg/dL [26], [42], [43].

Recurrent CDI was strongly associated with an increased likelihood of complicated CDI (OR = 2.7; 95% CI = 1.2–5.8 and OR = 4.1; 95% CI = 1.5–9.4, respectively) [44], [45], as well as exposure to particular treatments (Table 2). Ribotypes 018 and 056 were risk factors for complicated CDI (OR = 6.2; 95% CI = 1.3–23.8 and OR = 13.0; 95% CI = 1.1–148.3, respectively), in a pan-European study (Table 1) [46]. Ribotype 027 was not significantly associated with complicated CDI in multivariate analysis nor with indices of severity in other studies [47], [48], while _ENREF_48strains harbouring binary toxin gene were associated with complicated CDI in one study (OR = 5.9; 95% CI = 1.5–23.8) [49]. Other factors were associated with complicated CDI in one or two studies each (Table 2).

**Table 2:** Infrequent risk factors for complicated CDI and 30-day mortality.

Factor	OR/HR/RR (95% CI)
Complicated CDI	
Hospital-acquired CDI [42]	4.6 (2.4–8.6)
Severe diarrhoea [85]	3.6 (1.2–11.1)
Small bowel obstruction or ileus [41]	3.1 (1.0–9.4)
Recurrent CDI [44], [45]	2.7 (1.2–5.8), 4.1 (1.5–9.4)
Serum albumin <2.5g/dL [41]	3.4 (1.6–7.6)
Increase in C-reactive protein* [86]	1.15 (1.08–1.2)
Increase in procalcitonin level [47]	3.1 (1.5–6.3)
Abnormal abdominal CT-scan [41]	13.5 (5.7–32.1)
Confusion [44]	2.0 (1.05–3.8)
Abbreviated mental score <7 [87]	11.0 (2.3–58.8)
Endoscopy [87]	4.0 (1.2–14.9)
Tube feeding within prior 2 months [42]	2.4 (1.5–3.9)
Any operative therapy within prior 30 days [88]	3.5 (1.1–10.8)
Surgery in the previous two months [42]	0.6 (0.4–0.9)
Immunosuppression** [42]	2.3 (1.5–3.6)
Prior corticosteroid use [58]	2.1 (1.01–4.35)
Prior acid suppression use [58]	2.4 (1.2–4.8)
Prior intravenous immunoglobulin therapy [88]	8.9 (2.2–36.1)
Prior use of fluoroquinolones [43]	2.0 (0.98–4.1)
Use of exacerbating Abx after CDI [44]	3.0 (1.6–5.8)
30-day mortality	
Colectomy [73], [89]	0.2 (0.1–0.7); 40 (2.8–576.4)
Prolonged hospitalization before CDI (> 15 days) [90]	0.13 (0.03–0.6)
Hospital-acquired CDI [21]	1.9 (1.5–2.6)
ICU care [91]	2.8 (1.5–5.4)
Response failure to treatment [91]	3.9 (1.4–10.7)
Occult blood in stool [92]	0.32 (0.11–0.9)
Positive stool occult blood test [90]	6.3 (1.13–35.3)
Peak lactate ≥ 5 mmol/L [73]	12.4 (2.4–63.7)
Low peak day 12 anti-toxin A IgG [93]	0.97 (0.95–0.99)
Immunosuppression [89]	35.8 (2.8–464.5)
Immunosuppression *** for at least 1 month [73]	7.9 (2.3–27.2)
Any glucocorticoid use [94]	1.8 (1.62–1.98)

Abx = antibiotics. ICU = intensive care unit. IgG = immunoglobulin G.

*For each increment of 10 mg/mL.

^**^Chemotherapy, HIV infection, neutropenia, organ transplantation, or use of immunosuppressive drugs.

^***^ Systemic corticosteroids, leukaemia, lymphoma, organ transplant, or neutropenia.

#### 3. Risk factors for treatment failure (8 studies)

**Figure 6:**
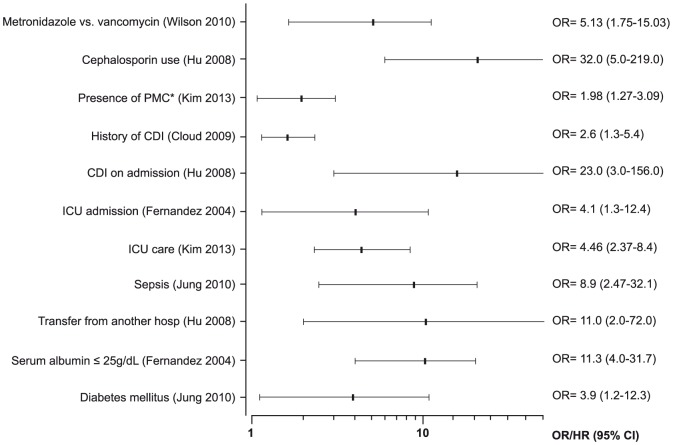
Forest plots of reported associations with treatment failure. *PMC = pseudomembranous colitis.

The definition of this outcome was heterogeneous, corresponding to a lack of improvement of symptoms after 5 to 10 days of the initial treatment (Table S2 and Table S4). Only need of intensive care was associated with treatment failure (mainly during metronidazole treatment) in more than one study (Figure 6). Increasing age (in decades OR = 1.14; 95% CI = 1.01–1.29) and increasing WBC in elderly patients (OR = 1.1; 95% CI = 1.0–1.2) were significant factors in one study each [50]–[52].

#### 4. Risk factors for mortality (30 studies)

Most included studies (73%) measured mortality within the 30-day interval after diagnosis, as per the current recommendations for CDI surveillance [1]. In the other studies, follow-up ranged between 14 [23], [53], 60 [43], and 90 days [51], [54], while nine studies did not specify any duration (Table S3 and Table S4).

**Figure 7:**
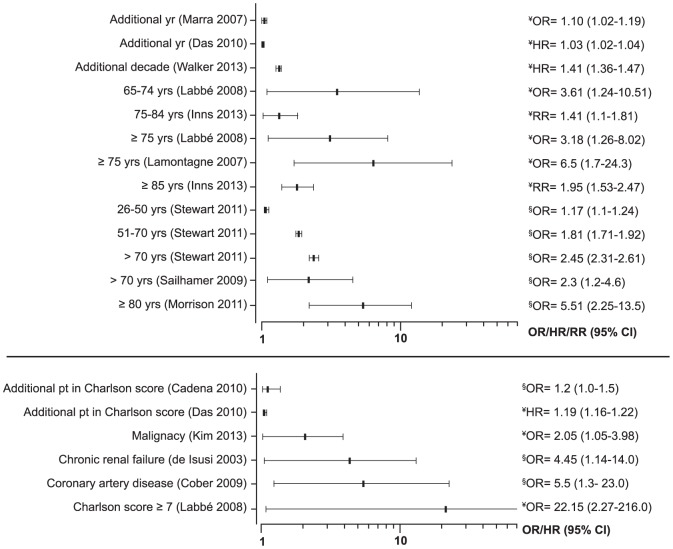
Forest plots of associations of age and co-morbidities with mortality. (¥≤30-day mortality; § >30-day).

**Figure 8:**
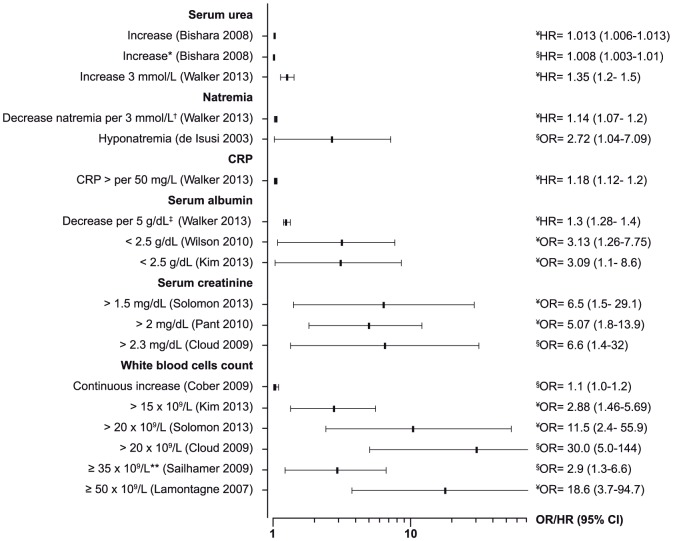
Forest plots of associations of blood tests with mortality. (¥≤30-day mortality; § >30-day)**.** *Increase in serum urea associated with 28-days and long-term mortality. †Original value: Sodium per 3 mmol/L higher <136; HR = 0.88 (0.83–0.93). **Leucocytosis: WBC≥35×10^9^/L or leucopenia: WBC<4×10^9^/L. ‡Original value: Albumin per 5 g/dL higher; HR = 0.74 (0.71–0.78).

Mortality, overall or due to CDI, was mainly associated with age (9 studies), underlying co-morbidities (6 studies) (Figure 7), and laboratory parameters (overall 11 studies): leucocytosis, increased serum urea, increased serum creatinine, elevated C-reactive protein, hypo-natremia and serum albumin (Figure 8). Ribotype 027 was associated with 30-day mortality in 5 studies with a relative risk ranging between 1.3 and 10.4 (Table 1) [21], [23], [55]–[57].

A severe CDI defined by two or more of age >60, leucocytosis, albumin <2.5 mg/dL or ICU admission almost doubled the risk of 90-day overall mortality after adjustment for co-morbidities (OR = 1.8; 95% CI = 1.2–2.6) [54]. Laboratory parameters were associated with all-cause 30-day mortality in one study each (Figure 8). High levels of WBC (>20×10^9^/L and ≥ 50×10^9^/L) were more strongly associated with death than with complicated CDI (Figure 5) [43], [54]. Other factors associated with 30-day mortality reported in one study or two studies are shown in Table 2. Continuous increase in WBC was associated with 90-day mortality in one study [51], and prior exposure to acid suppression therapy was associated with mortality in one study where the delay was not reported [58]. In one study [43], death with CDI as contributor was associated with WBC >20×10^9 ^cells/L, serum creatinine >2.3 mg/dL and exposure to fluoroquinolones within 60 days.

Six other studies were conducted on patients requiring surgical treatment for CDI (colectomy or hemicolectomy) [24], [59]–[64]. Risk factors associated with mortality were older age [62], [63], high leucocytosis [61], preoperative hypo-albuminaemia [61], [62], preoperative increase in serum lactate [62], and duration of treatment [59].

### Risk of bias assessment

**Figure 9:**
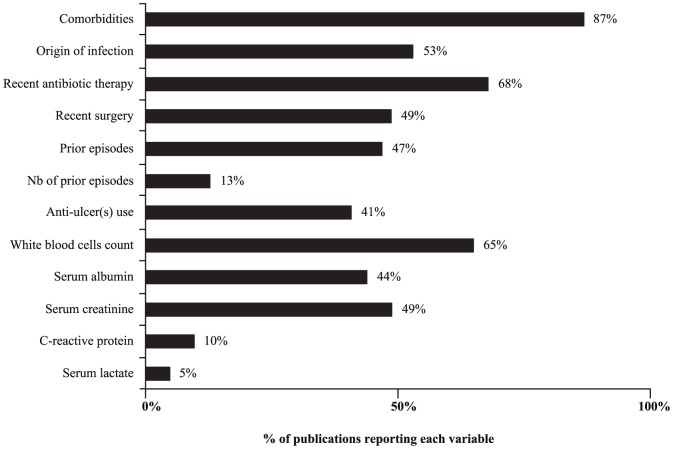
Quality evaluation of included studies (n = 68) according to reported clinical data.

Almost all studies reported age and gender (96%) of their study populations, and the majority reported confirmed cases of CDI and co-morbidities (90% and 87% respectively). Only half (53%) of studies reported the site of acquisition (nosocomial versus community-acquired), recent surgical or other procedures (49%) and previous episodes of CDI (47%) (Figure 9). One third of included studies (n = 23) provided strain typing of *C. difficile*, but only 14 included the strain type in multivariate analyses. The association with outcomes and the period of data collection are presented in Table 1. Recent antibiotic (46; 68%) and immunosuppressive therapies (38; 56%) were frequently reported. Very few studies reported measures of serum lactate, C-reactive protein, and procalcitonin.

Among studies on mortality alone, only half of them reported the site of acquisition of CDI, as did around half of studies on recurrence and 64% on complicated CDI. Having experienced any previous episode of CDI was reported in only 53% of studies on recurrence and mortality (56%), and in only one third of studies on complicated CDI and multiple outcomes.

As for statistical analyses, the median number of variables in the final model (including statistically significant variables and all adjustments) was 7 (range 2–18). The median number of EPV was only 6.6 (range 0.6–430) (Table S1–S4). Only one-third (23; 34%) of studies had 10 EPV or more.

## Discussion

This review is the largest on unfavourable outcomes of CDI (68 studies), based on publications from 1978 until 2013, and the first to gather risk factors for CDI-related complications. It also represents an important update about risk factors for recurrence. Publications were subjected to two stages of screening before final inclusion and a quality control process was performed during all steps of the review. Studies with univariate comparisons were left out, as it would be irrelevant to consider clinical parameters, co-morbidities and medications as independent factors when confounding and interaction were not addressed through multivariate analyses. A previous review of studies with a sample size ≥100 patients [14], used mortality as a keyword rather than an outcome for the search as suggested by Population Intervention Comparison Outcome (PICO) frameworks [17], which could have restricted the number of retrieved studies. Moreover, only inclusion criteria and study characteristics were used as markers of acceptable quality [14]. However, according to PRISMA guidelines [17], we qualitatively assessed the risk of bias across studies, but did not assess it individually. Recent reviews showed a lack in relevant tools such as scales, checklists, or quality criteria for observational studies [65]–[67]. Available tools involve a subjective assessment of risk of bias, leading to inconsistent validity and reliability [65], and are more appropriate for interventional trials. Consequently, we assessed the quality of studies according to standard methodologies, and used an objective statement of bias through the measurement and reporting of relevant data.

As in a previous review, continued use of antibiotics, concomitant anti-ulcer medication and older age were risk factors for recurrence [13]. Concomitant use of antibiotics and PPIs have an additive effect on increasing susceptibility to CDI [68], [69], which could explain the higher risk of recurrence. However, multivariate adjustment on use of antibiotics or PPIs was performed in only four [20], [32], [34], [35], of the nine studies where those variables were associated with recurrence.

Several other limitations were observed across included studies. Small sample sizes (median 128) led to wide confidence intervals in estimations of relative risks. Adjustment for confounders was not always clear in included publications even if this represents an important factor for the validity of results [70]. Only 14 studies included the strain type as an independent variable in multivariate analysis. We could not use the year 2002 as a cut-off date for the introduction of NAP1/BI/027 strain [8], [42], because the timing of its introduction varied considerably between countries and regions, and several studies collected data over long periods overlapping this date (Table 1).

### Why we could not perform a meta-analysis

The quality of a meta-analysis depends heavily on the individual quality of pooled data. Hence, multiple methodological gaps and substantial heterogeneity across included studies would have led to an inappropriate meta-analysis. Most studies were conducted retrospectively with data gathered from medical charts and/or electronic databases. Although minimizing recall biases, this methodology is often hampered by missing data. Missing data in the original publications, mainly observational studies, was an important limitation for the estimation of the effect of risk factors. For instance, while previous episodes are likely to be a risk factor for recurrence [46], [71], only half of included studies reported any previous episode of CDI and 13% reported the number of previous episodes. Except for mortality, the definition of the outcome, particularly complications, and the duration of follow-up differed between studies. Most studies accounted for all-cause mortality within their definition of complicated CDI. Risk of 30-day mortality ranged between 8 and 31% in studies having death as the main outcome where all CDI cases were considered, while four studies conducted on patients enrolled in ICU [53], [72]–[74] reported risk of mortality ranging from 25 to 53%. In studies of patients who underwent a colectomy, where rates of mortality were particularly high, data were collected over 7 to 13 years, and except for one study [24], sample sizes were very small (n = 13–130). All of those studies recommended early surgery to prevent organ failure and to decrease mortality. Thus variations in overall mortality reflected either the selection of the sickest patients, causes of death unrelated to CDI, or perhaps differences in the pathogenicity of local *C. difficile* strains. Treatment failure was considered separately from complications in 8 studies, but without any common risk factors.

Poor reporting and considerable heterogeneity was noted in the diagnostic tests which defined cases of CDI, these tests differing in sensitivity and specificity [75], [76]. Diagnosis was mostly confirmed with EIAs (toxin A alone, or A+B) despite their low sensitivity [77]. Only 33% of the studies used diagnostic tests of higher sensitivity and specificity: CTA in 19% (n = 12), PCR in 9% (n = 6), and toxigenic culture in 5% (n = 3). As a consequence, studies using EIAs might have included sicker patients, while those based on PCR might have included patients merely colonized with *C. difficile* presenting an episode of diarrhea unrelated to this pathogen, and patients at an early stage of the disease [78], [79]. In addition, the methods used for strain typing and the definition of some variables such as the scores for co-morbidities and severity of CDI were highly heterogeneous. The cut-off points considered for leucocytosis varied between 12 and >50x10^9^/L. A similar wide range was observed in the creatinine level. Evaluation of laboratory parameters as predictors was limited to frequently ordered tests: less than 10% of studies reported levels of serum lactate or C-reactive protein or procalcitonin.

### Current guidelines for case-management

Currently, two American guidelines define patients with severe CDI (for whom the initial treatment should be vancomycin, a drug thought to lower the risk of complications) as those with a leucocytosis (WBC >15×10^9^/L) and/or a creatinine >1.5 times the baseline [80], and with WBC >15×10^9^/L plus a serum albumin <3 g/dl or abdominal tenderness [75]. European guidelines use the same cut-offs of leucocytosis and creatinine, but include many other clinical, radiologic or laboratory criteria in their definition of severe CDI for whom vancomycin is recommended [81]. Whether age over 65 years or co-morbidities should by themselves be a criterion for severity is left to the discretion of the attending physician [81]. A recent meta-analysis on the treatment of recurrent CDI provided moderate evidence on the efficacy of available treatments [82]. Despite low to moderate evidence, vancomycin combined with metronidazole was recommended for severe and complicated cases [75]. Thus, while the three laboratory parameters (leucocytes, serum creatinine and albumin) identified by our systematic review are incorporated within current guidelines, older age remains to be properly addressed.

## Conclusions

Currently available studies about risk factors and clinical parameters allowing the prediction of unfavourable outcomes in CDI are heterogeneous. Older age, antibiotics after the diagnosis of CDI, use of PPIs, and strain type are the most frequent risk factors for recurrence. Older age, leucocytosis, renal failure and underlying co-morbidities are frequent risk factors for complicated CDI, including mortality in many cases. As for mortality alone, in addition to age, it seems to be associated with co-morbidities, decreased serum albumin, leucocytosis, increased serum creatinine and/or urea and ribotype 027 (30-day mortality). Laboratory parameters used in American and European guidelines (high leucocytosis, acute renal failure) are adequate to define patients at risk of complications. The patient’s age should be a key factor in the management of CDI. It would seem advisable for future iterations of these guidelines to incorporate age within their decisional algorithms, so as to offer to the elderly potentially more effective drugs such as vancomycin or fidaxomicin.

## Addendum

While this manuscript was being evaluated, a study documented an association between low levels of vitamin D and increasing severity of CDI (defined as an abnormal CT scan and fulminant colitis) [83], and another one reported an association between low vitamin D levels and a composite outcome of all-cause 30-day mortality and/or recurrence [84]. Both were small studies and further work is necessary to define whether or not vitamin D deficiency is genuinely associated with adverse outcomes of CDI.

## Supporting Information

Table S1 **Characteristics of included studies addressing risk factors for recurrence [20], [25], [27]–[40], [71], [95], [96].**
(PDF)

Table S2 **Characteristics of included studies addressing risk factors for complicated CDI and treatment failure [41], [42], [44], [45], [47], [48], [50], [85]–[88], [97], [98].**
(PDF)

Table S3 **Characteristics of included studies addressing risk factors for mortality considered alone [21]–[24], [53], [55]–[57], [59]–[63], [72]–[74], [89], [90], [92], [94], [99]–[101].**
(PDF)

Table S4 **Characteristics of included studies addressing risk factors for multiple outcomes [26], [43], [46], [49], [51], [52], [54], [58], [91], [93], [102]–[104].**
(PDF)

Checklist S1 **PRISMA checklist.**
(DOC)

Text S1 **Electronic search: databases and keywords.**
(PDF)

## References

[1] McDonald LC, Coignard B, Dubberke E, Song X, Horan T, et al (2007) Recommendations for surveillance of *Clostridium difficile*-associated disease. Infect Control Hosp Epidemiol 28: 140–5. 17265394 10.1086/511798

[2] Aslam S, Hamill RJ, Musher DM (2005) Treatment of *Clostridium difficile*-associated disease: old therapies and new strategies. Lancet Infect Dis 5: 549–57. 16122678 10.1016/S1473-3099(05)70215-2

[3] Rupnik M, Wilcox MH, Gerding DN (2009) *Clostridium difficile* infection: new developments in epidemiology and pathogenesis. Nat Rev Microbiol 7: 526–36. 19528959 10.1038/nrmicro2164

[4] Nelson RL, Kelsey P, Leeman H, Meardon N, Patel H, et al (2011) Antibiotic treatment for *Clostridium difficile*-associated diarrhea in adults. Cochrane Database Syst Rev 9: CD004610. 10.1002/14651858.CD004610.pub4 21901692

[5] Hensgens MP, Goorhuis A, Dekkers OM, Kuijper EJ (2012) Time interval of increased risk for *Clostridium difficile* infection after exposure to antibiotics. J Antimicrob Chemother 67: 742–8. 22146873 10.1093/jac/dkr508

[6] Thomas C, Stevenson M, Riley TV (2003) Antibiotics and hospital-acquired *Clostridium difficile*-associated diarrhoea: a systematic review. J Antimicrob Chemother 51: 1339–50. 12746372 10.1093/jac/dkg254

[7] McFarland LV (2009) Renewed interest in a difficult disease: *Clostridium difficile* infections—epidemiology and current treatment strategies. Curr Opin Gastroenterol 25: 24–35. 19114771 10.1097/MOG.0b013e32831da7c4

[8] Kuijper EJ, Coignard B, Tull P (2006) Emergence of *Clostridium difficile*-associated disease in North America and Europe. Clin Microbiol Infect 12 Suppl 62–18. 10.1111/j.1469-0691.2006.01580.x 16965399

[9] Louie TJ, Miller MA, Mullane KM, Weiss K, Lentnek A, et al (2011) Fidaxomicin versus vancomycin for *Clostridium difficile* infection. N Engl J Med 364: 422–31. 21288078 10.1056/NEJMoa0910812

[10] Lowy I, Molrine DC, Leav BA, Blair BM, Baxter R, et al (2010) Treatment with monoclonal antibodies against *Clostridium difficile* toxins. N Engl J Med 362: 197–205. 20089970 10.1056/NEJMoa0907635

[11] Drekonja DM, Butler M, MacDonald R, Bliss D, Filice GA, et al (2011) Comparative effectiveness of *Clostridium difficile* treatments: a systematic review. Ann Intern Med 155: 839–47. 22184691 10.7326/0003-4819-155-12-201112200-00007

[12] Abou Chakra CN, Pepin J, Valiquette L (2012) Prediction tools for unfavourable outcomes in *Clostridium difficile* infection: a systematic review. PLoS One 7: e30258. 22291926 10.1371/journal.pone.0030258 PMC3265469

[13] Garey KW, Sethi S, Yadav Y, DuPont HL (2008) Meta-analysis to assess risk factors for recurrent *Clostridium difficile* infection. J Hosp Infect70: 298–304. 10.1016/j.jhin.2008.08.012 18951661

[14] Bloomfield MG, Sherwin JC, Gkrania-Klotsas E (2012) Risk factors for mortality in *Clostridium difficile* infection in the general hospital population: a systematic review. J Hosp Infect 82(1): 1–12. 22727824 10.1016/j.jhin.2012.05.008

[15] Karas JA, Enoch DA, Aliyu SH (2010) A review of mortality due to *Clostridium difficile* infection. J Infect 61: 1–8. 20361997 10.1016/j.jinf.2010.03.025

[16] Mitchell BG, Gardner A (2012) Mortality and *Clostridium difficile* infection: a review. Antimicrob Resist Infect Control 1: 20. 22958425 10.1186/2047-2994-1-20 PMC3533881

[17] Moher D, Liberati A, Tetzlaff J, Altman DG (2009) Preferred reporting items for systematic reviews and meta-analyses: the PRISMA Statement. Open Med 3: e123–e30. 21603045 PMC3090117

[18] Laupacis A, Sekar N, Stiell IG (1997) Clinical prediction rules. A review and suggested modifications of methodological standards. JAMA 277: 488–94. 9020274

[19] Peduzzi P, Concato J, Kemper E, Holford TR, Feinstein AR (1996) A simulation study of the number of events per variable in logistic regression analysis. J Clin Epidemiol 49: 1373–9. 8970487 10.1016/s0895-4356(96)00236-3

[20] Linsky A, Gupta K, Lawler EV, Fonda JR, Hermos JA (2010) Proton pump inhibitors and risk for recurrent *Clostridium difficile* infection. Arch Intern Med 170: 772–8. 20458084 10.1001/archinternmed.2010.73

[21] Inns T, Gorton R, Berrington A, Sails A, Lamagni T, et al (2013) Effect of ribotype on all-cause mortality following *Clostridium difficile* infection. J Hosp Infect 84: 235–41. 23759667 10.1016/j.jhin.2013.04.008

[22] Stewart DB, Hollenbeak CS (2011) *Clostridium difficile* Colitis: Factors Associated with Outcome and Assessment of Mortality at a National Level. J Gastrointest Surg 15: 1548–55. 21720924 10.1007/s11605-011-1615-6

[23] Walker AS, Eyre DW, Wyllie DH, Dingle KE, Griffiths D, et al (2013) Relationship between bacterial strain type, host biomarkers, and mortality in *Clostridium difficile* infection. Clin Infect Dis 56: 1589–600. 23463640 10.1093/cid/cit127 PMC3641870

[24] Halabi WJ, Nguyen VQ, Carmichael JC, Pigazzi A, Stamos MJ, et al (2013) *Clostridium difficile* Colitis in the United States: A Decade of Trends, Outcomes, Risk Factors for Colectomy, and Mortality after Colectomy. J Am Coll Surg 217(5): 802–12. 24011436 10.1016/j.jamcollsurg.2013.05.028

[25] Eyre DW, Walker AS, Wyllie D, Dingle KE, Griffiths D, et al (2012) Predictors of First Recurrence of *Clostridium difficile* Infection: Implications for Initial Management. Clin Infect Dis 55: S77–87. 22752869 10.1093/cid/cis356 PMC3388024

[26] Pepin J, Routhier S, Gagnon S, Brazeau I (2006) Management and outcomes of a first recurrence of *Clostridium difficile*-associated disease in Quebec, Canada. Clin Infect Dis 42: 758–64. 16477549 10.1086/501126

[27] Freedberg DE, Salmasian H, Friedman C, Abrams JA (2013) Proton Pump Inhibitors and Risk for Recurrent *Clostridium difficile* Infection Among Inpatients. Am J Gastroenterol 108(11): 1794–801. 24060760 10.1038/ajg.2013.333 PMC3966060

[28] McFarland LV, Surawicz CM, Rubin M, Fekety R, Elmer GW, et al (1999) Recurrent *Clostridium difficile* disease: epidemiology and clinical characteristics. Infect Control Hosp Epidemiol 20: 43–50. 9927265 10.1086/501553

[29] Lavergne V, Beausejour Y, Pichette G, Ghannoum M, Su SH (2013) Lymphopenia as a novel marker of *Clostridium difficile* infection recurrence. J Infect 66: 129–35. 23178420 10.1016/j.jinf.2012.11.001

[30] Kim JW, Lee KL, Jeong JB, Kim BG, Shin S, et al (2010) Proton pump inhibitors as a risk factor for recurrence of Clostridium-difficile-associated diarrhea. World J Gastroenterol16 (28): 3573–77. 10.3748/wjg.v16.i28.3573 PMC2909558 20653067

[31] Pepin J, Alary ME, Valiquette L, Raiche E, Ruel J, et al (2005) Increasing risk of relapse after treatment of *Clostridium difficile* colitis in Quebec, Canada. Clin Infect Dis 40: 1591–7. 15889355 10.1086/430315

[32] Kyne L, Warny M, Qamar A, Kelly CP (2001) Association between antibody response to toxin A and protection against recurrent *Clostridium difficile* diarrhoea. Lancet 357: 189–93. 11213096 10.1016/S0140-6736(00)03592-3

[33] Petrella LA, Sambol SP, Cheknis A, Nagaro K, Kean Y, et al (2012) Decreased cure and increased recurrence rates for *Clostridium difficile* infection caused by the epidemic *C. difficile* BI strain. Clin Infect Dis 55: 351–7. 22523271 10.1093/cid/cis430 PMC3491778

[34] Drekonja DM, Amundson WH, Decarolis DD, Kuskowski MA, Lederle FA, et al (2011) Antimicrobial Use and Risk for Recurrent *Clostridium difficile* Infection. Am J Med Nov 124(11): 1081.e1–7. 10.1016/j.amjmed.2011.05.032 21944159

[35] Kim YG, Graham DY, Jang BI (2012) Proton pump inhibitor use and recurrent *Clostridium difficile*-associated disease: a case-control analysis matched by propensity score. J Clin Gastroenterol 46: 397–400. 22298089 10.1097/MCG.0b013e3182431d78

[36] Louie TJ, Miller MA, Crook DW, Lentnek A, Bernard L, et al (2013) Effect of age on treatment outcomes in *Clostridium difficile* infection. J Am Geriatr Soc 61: 222–30. 23379974 10.1111/jgs.12090

[37] Stewart DB, Berg A, Hegarty J (2013) Predicting recurrence of *C. difficile* colitis using bacterial virulence factors: binary toxin is the key. J Gastrointest Surg 17: 118-24; discussion p 24–5. 10.1007/s11605-012-2056-6 23086451

[38] Marsh JW, Arora R, Schlackman JL, Shutt KA, Curry SR, et al (2012) Association of relapse of *Clostridium difficile* disease with BI/NAP1/027. J Clin Microbiol 50: 4078–82. 23052318 10.1128/JCM.02291-12 PMC3502988

[39] Do AN, Fridkin SK, Yechouron A, Banerjee SN, Killgore GE, et al (1998) Risk factors for early recurrent *Clostridium difficile*-associated diarrhea. Clin Infect Dis 26: 954–9. 9564482 10.1086/513952

[40] Garey KW, Jiang ZD, Ghantoji S, Tam VH, Arora V, et al (2010) A Common Polymorphism in the Interleukin-8 Gene Promoter Is Associated with an Increased Risk for Recurrent *Clostridium difficile* Infection. Clin Infect Dis 51: 1406–10. 21058913 10.1086/657398

[41] Henrich TJ, Krakower D, Bitton A, Yokoe DS (2009) Clinical risk factors for severe *Clostridium difficile*-associated disease. Emerg Infect Dis 15: 415–22. 19239754 10.3201/eid1503.080312 PMC2681109

[42] Pepin J, Valiquette L, Alary ME, Villemure P, Pelletier A, et al (2004) *Clostridium difficile*-associated diarrhea in a region of Quebec from 1991 to 2003: a changing pattern of disease severity. CMAJ 171: 466–72. 15337727 10.1503/cmaj.1041104 PMC514643

[43] Cloud J, Noddin L, Pressman A, Hu M, Kelly C (2009) *Clostridium difficile* strain NAP-1 is not associated with severe disease in a nonepidemic setting. Clin Gastroenterol Hepatol 7 : 868–73 e2. 10.1016/j.cgh.2009.05.018 19465153

[44] Manek K, Williams V, Callery S, Daneman N (2011) Reducing the risk of severe complications among patients with *Clostridium difficile* infection. Can J Gastroenterol 25: 368–72. 21876858 10.1155/2011/153020 PMC3174077

[45] Andrews CN, Raboud J, Kassen BO, Enns R (2003) *Clostridium difficile*-associated diarrhea: predictors of severity in patients presenting to the emergency department. Can J Gastroenterol 17: 369–73. 12813602 10.1155/2003/723471

[46] Bauer MP, Notermans DW, van Benthem BH, Brazier JS, Wilcox MH, et al (2011) *Clostridium difficile* infection in Europe: a hospital-based survey. Lancet 377: 63–73. 21084111 10.1016/S0140-6736(10)61266-4

[47] Rao K, Walk ST, Micic D, Chenoweth E, Deng L, et al (2013) Procalcitonin levels associate with severity of *Clostridium difficile* infection. PLoS One 8: e58265. 23505476 10.1371/journal.pone.0058265 PMC3591407

[48] Walk ST, Micic D, Jain R, Lo ES, Trivedi I, et al (2012) *Clostridium difficile* ribotype does not predict severe infection. Clin Infect Dis 55: 1661–8. 22972866 10.1093/cid/cis786 PMC3501335

[49] Soes LM, Brock I, Persson S, Simonsen J, Pribil Olsen KE, et al (2012) Clinical features of *Clostridium difficile* infection and molecular characterization of the isolated strains in a cohort of Danish hospitalized patients. Eur J Clin Microbiol Infect Dis 31: 185–92. 21744281 10.1007/s10096-011-1292-0

[50] Hu MY, Maroo S, Kyne L, Cloud J, Tummala S, et al (2008) A prospective study of risk factors and historical trends in metronidazole failure for *Clostridium difficile* infection. Clin Gastroenterol Hepatol 6: 1354–60. 19081526 10.1016/j.cgh.2008.06.024 PMC2644212

[51] Cober ED, Malani PN (2009) *Clostridium difficile* infection in the "oldest" old: clinical outcomes in patients aged 80 and older. J Am Geriatr Soc 57: 659–62. 19392957 10.1111/j.1532-5415.2009.02182.x

[52] Khanna S, Aronson SL, Kammer PP, Baddour LM, Pardi DS (2012) Gastric acid suppression and outcomes in *Clostridium difficile* infection: a population-based study. Mayo Clin Proc 87: 636–42. 22766083 10.1016/j.mayocp.2011.12.021 PMC3538480

[53] Marra AR, Edmond MB, Wenzel RP, Bearman GM (2007) Hospital-acquired *Clostridium difficile*-associated disease in the intensive care unit setting: epidemiology, clinical course and outcome. BMC Infect Dis 7: 42. 17517130 10.1186/1471-2334-7-42 PMC1888698

[54] Cadena J, Thompson GR, Patterson JE, Nakashima B, Owens A, et al (2010) Clinical predictors and risk factors for relapsing *Clostridium difficile* infection. Am J MedSci339 (4): 350–55. 10.1097/MAJ.0b013e3181d3cdaa 20224312

[55] Labbe AC, Poirier L, Maccannell D, Louie T, Savoie M, et al (2008) *Clostridium difficile* infections in a Canadian tertiary care hospital before and during a regional epidemic associated with the BI/NAP1/027 strain. Antimicrob Agents Chemother 52: 3180–7. 18573937 10.1128/AAC.00146-08 PMC2533448

[56] Huttunen R, Vuento R, Syrjanen J, Tissari P, Aittoniemi J (2012) Case fatality associated with a hypervirulent strain in patients with culture-positive *Clostridium difficile* infection: a retrospective population-based study. Int J Infect Dis 16: e532–5. 22572612 10.1016/j.ijid.2012.02.019

[57] Goorhuis A, Debast SB, Dutilh JC, van Kinschot CM, Harmanus C, et al (2011) Type-specific risk factors and outcome in an outbreak with 2 different *Clostridium difficile* types simultaneously in 1 hospital. Clin Infect Dis 53: 860–9. 21914851 10.1093/cid/cir549

[58] Morrison RH, Hall NS, Said M, Rice T, Groff H, et al (2011) Risk Factors Associated With Complications and Mortality in Patients With *Clostridium difficile* Infection. . Clin Infect Dis. 53(9): 860–9. 21976459 10.1093/cid/cir668

[59] Byrn JC, Maun DC, Gingold DS, Baril DT, Ozao JJ, et al (2008) Predictors of mortality after colectomy for fulminant *Clostridium difficile* colitis. Arch Surg 143: 150–4 discussion 55. 18283139 10.1001/archsurg.2007.46

[60] Perera AD, Akbari RP, Cowher MS, Read TE, McCormick JT, et al (2010) Colectomy for fulminant *Clostridium difficile* colitis: Predictors of mortality. Am Surg 76 (4): 418–21. 10.1177/000313481007600421 20420254

[61] Markelov A, Livert D, Kohli H (2011) Predictors of Fatal Outcome after Colectomy for Fulminant *Clostridium difficile* Colitis: A 10-Year Experience. Am Surg 77: 977–80. 21944509 10.1177/000313481107700813

[62] Pepin J, Vo TT, Boutros M, Marcotte E, Dial S, et al (2009) Risk factors for mortality following emergency colectomy for fulminant *Clostridium difficile* infection. Dis Colon Rectum 52 (3): 400–05. 10.1007/DCR.0b013e31819a69aa 19333038

[63] Seder CW, Villalba MR, Robbins J, Ivascu FA, Carpenter CF, et al (2009) Early colectomy may be associated with improved survival in fulminant *Clostridium difficile* colitis: an 8-year experience. Am J Surg197: 302–7. 10.1016/j.amjsurg.2008.11.001 19245905

[64] Hall JF, Berger D (2008) Outcome of colectomy for *Clostridium difficile* colitis: a plea for early surgical management. Am J Surg 196: 384–8. 18519126 10.1016/j.amjsurg.2007.11.017

[65] Shamliyan T, Kane RL, Dickinson S (2010) A systematic review of tools used to assess the quality of observational studies that examine incidence or prevalence and risk factors for diseases. J Clin Epidemiol 63: 1061–70. 20728045 10.1016/j.jclinepi.2010.04.014

[66] Sanderson S, Tatt ID, Higgins JP (2007) Tools for assessing quality and susceptibility to bias in observational studies in epidemiology: a systematic review and annotated bibliography. Int J Epidemiol 36: 666–76. 17470488 10.1093/ije/dym018

[67] Herbison P, Hay-Smith J, Gillespie WJ (2006) Adjustment of meta-analyses on the basis of quality scores should be abandoned. J Clin Epidemiol 59: 1249–56. 17098567 10.1016/j.jclinepi.2006.03.008

[68] Stevens V, Dumyati G, Brown J, Wijngaarden E (2011) Differential risk of *Clostridium difficile* infection with proton pump inhibitor use by level of antibiotic exposure. Pharmacoepidemiol Drug Saf 20: 1035–42. 21833992 10.1002/pds.2198

[69] Bavishi C, Dupont HL (2011) Systematic review: the use of proton pump inhibitors and increased susceptibility to enteric infection. Aliment Pharmacol Ther 34: 1269–81. 21999643 10.1111/j.1365-2036.2011.04874.x

[70] Egger M, Schneider M, Davey Smith G (1998) Spurious precision? Meta-analysis of observational studies. BMJ 316: 140–4. 9462324 10.1136/bmj.316.7125.140 PMC2665367

[71] Fekety R, McFarland LV, Surawicz CM, Greenberg RN, Elmer GW, et al (1997) Recurrent *Clostridium difficile* diarrhea: Characteristics of and risk factors for patients enrolled in a prospective, randomized, double-blinded trial. Clin Infect Dis 24 (3): 324–33. 10.1093/clinids/24.3.324 9114180

[72] Kenneally C, Rosini JM, Skrupky LP, Doherty JA, Hollands JM, et al (2007) Analysis of 30-day mortality for *Clostridium difficile*-associated disease in the ICU setting. Chest 132: 418–24. 17573523 10.1378/chest.07-0202

[73] Lamontagne F, Labbe AC, Haeck O, Lesur O, Lalancette M, et al (2007) Impact of emergency colectomy on survival of patients with fulminant *Clostridium difficile* colitis during an epidemic caused by a hypervirulent strain. Ann Surg 245: 267–72. 17245181 10.1097/01.sla.0000236628.79550.e5 PMC1876996

[74] Sailhamer EA, Carson K, Chang Y, Zacharias N, Spaniolas K, et al (2009) Fulminant *Clostridium difficile* colitis: patterns of care and predictors of mortality. Arch Surg 144: 433–9 discussion 39–40. 19451485 10.1001/archsurg.2009.51

[75] Surawicz CM, Brandt LJ, Binion DG, Ananthakrishnan AN, Curry SR et al. (2013) Guidelines for diagnosis, treatment, and prevention of *Clostridium difficile* infections. Am J Gastroenterol 108: 478–98; quiz 99. 10.1038/ajg.2013.4 23439232

[76] Planche T, Aghaizu A, Holliman R, Riley P, Poloniecki J, et al (2008) Diagnosis of *Clostridium difficile* infection by toxin detection kits: a systematic review. Lancet Infect Dis 8: 777–84. 18977696 10.1016/S1473-3099(08)70233-0

[77] Stanley JD, Bartlett JG, Dart BWt, Ashcraft JH (2013) *Clostridium difficile* infection. Curr Probl Surg 50: 302–37. 23764494 10.1067/j.cpsurg.2013.02.004

[78] Longtin Y, Trottier S, Brochu G, Paquet-Bolduc B, Garenc C, et al (2013) Impact of the type of diagnostic assay on *Clostridium difficile* infection and complication rates in a mandatory reporting program. Clin Infect Dis 56: 67–73. 23011147 10.1093/cid/cis840

[79] de Jong E, de Jong AS, Bartels CJ, van der Rijt-van den Biggelaar C, Melchers WJ, et al (2012) Clinical and laboratory evaluation of a real-time PCR for *Clostridium difficile* toxin A and B genes. Eur J Clin Microbiol Infect Dis 31: 2219–25. 22327373 10.1007/s10096-012-1558-1 PMC3418502

[80] Cohen SH, Gerding DN, Johnson S, Kelly CP, Loo VG, et al (2010) Clinical practice guidelines for *Clostridium difficile* infection in adults: 2010 update by the society for healthcare epidemiology of America (SHEA) and the infectious diseases society of America (IDSA). Infect Control Hosp Epidemiol 31: 431–55. 20307191 10.1086/651706

[81] Bauer MP, Kuijper EJ, van Dissel JT (2009) European Society of Clinical Microbiology and Infectious Diseases (ESCMID): treatment guidance document for *Clostridium difficile* infection (CDI). Clin Microbiol Infect 15: 1067–79. 19929973 10.1111/j.1469-0691.2009.03099.x

[82] O'Horo JC, Jindai K, Kunzer B, Safdar N (2013) Treatment of recurrent *Clostridium difficile* infection: a systematic review. Infection 42(1): 43–59. 23839210 10.1007/s15010-013-0496-x PMC3934353

[83] van der Wilden GM, Fagenholz PJ, Velmahos GC, Quraishi SA, Schipper IB et al. (2014) Vitamin D Status and Severity of *Clostridium difficile* Infections: A Prospective Cohort Study in Hospitalized Adults. J Parenter Enteral Nutr Jan 9 [Epub ahead of print] 10.1177/0148607113519129 24408036

[84] Wang WJ, Gray S, Sison C, Arramraju S, John BK, et al (2014) Low vitamin D level is an independent predictor of poor outcomes in *Clostridium difficile*-associated diarrhea. Therap Adv Gastroenterol 7: 14–9. 10.1177/1756283X13502838 PMC3871280 24381644

[85] Wenisch JM, Schmid D, Kuo HW, Simons E, Allerberger F, et al (2012) Hospital-acquired *Clostridium difficile* infection: determinants for severe disease. Eur J Clin Microbiol Infect Dis 31: 1923–30. 22210266 10.1007/s10096-011-1522-5

[86] Hardt C, Berns T, Treder W, Domoulin FL (2008) Univariate and multivariate analysis of risk factors for severe *Clostridium difficile*-associated diarrhoea: Importance of co-morbidity and serum C-reactive protein. World J Gastroenterol14 (27): 4338–41. 10.3748/wjg.14.4338 PMC2731185 18666322

[87] Kyne L, Merry C, O'Connell B, Kelly A, Keane C, et al (1999) Factors associated with prolonged symptoms and severe disease due to *Clostridium difficile* . Age Ageing 28: 107–13. 10350405 10.1093/ageing/28.2.107

[88] Greenstein AJ, Byrn JC, Zhang LP, Swedish KA, Jahn AE, et al (2008) Risk factors for the development of fulminant *Clostridium difficile* colitis. Surgery 143: 623–9. 18436010 10.1016/j.surg.2007.12.008

[89] Jansen A, Kleinkauf N, Weiss B, Zaiss NH, Witte W, et al (2010) Emergence of *Clostridium difficile* ribotype 027 in Germany: Epidemiological and clinical characteristics. Z Gastroenterol 48 (9): 1120–25. 10.1055/s-0029-1245269 20839161

[90] Khan FY, Abu-Khattab M, Anand D, Baager K, Alaini A, et al (2012) Epidemiological features of *Clostridium difficile* infection among inpatients at Hamad General Hospital in the state of Qatar, 2006–2009. Travel Med Infect Dis 10: 179–85. 22800937 10.1016/j.tmaid.2012.06.004

[91] Kim ES, Kim YJ, Park CW, Cho KB, Jang BK, et al (2013) Response failure to the treatment of *Clostridium difficile* infection and its impact on 30-day mortality. Hepatogastroenterology 60: 543–8. 23108084 10.5754/hge12730

[92] Bishara J, Peled N, Pitlik S, Samra Z (2008) Mortality of patients with antibiotic-associated diarrhoea: the impact of *Clostridium difficile* . J Hosp Infect 68: 308–14. 18353491 10.1016/j.jhin.2008.01.033

[93] Solomon K, Martin AJ, O'Donoghue C, Chen X, Fenelon L, et al (2013) Mortality in patients with *Clostridium difficile* infection correlates with host pro-inflammatory and humoral immune responses. J Med Microbiol 62: 1453–60. 23722431 10.1099/jmm.0.058479-0

[94] Das R, Feuerstadt P, Brandt LJ (2010) Glucocorticoids are associated with increased risk of short-term mortality in hospitalized patients with *Clostridium difficile*-associated disease. Am J Gastroenterol 105: 2040–9. 20389295 10.1038/ajg.2010.142

[95] Choi HK, Kim KH, Lee SH, Lee SJ (2011) Risk Factors for Recurrence of *Clostridium difficile* Infection: Effect of Vancomycin-resistant Enterococci Colonization. J Korean Med Sci 26: 859–64. 21738336 10.3346/jkms.2011.26.7.859 PMC3124713

[96] Shakov R, Salazar RS, Kagunye SK, Baddoura WJ, DeBari VA (2011) Diabetes mellitus as a risk factor for recurrence of *Clostridium difficile* infection in the acute care hospital setting. Am J Infect Control 39: 194–8. 21349600 10.1016/j.ajic.2010.08.017

[97] Gujja D, Friedenberg FK (2009) Predictors of serious complications due to *Clostridium difficile* infection. Aliment Pharmacol Ther 29: 635–42. 19077106 10.1111/j.1365-2036.2008.03914.x

[98] Fernandez A, Anand G, Friedenberg F (2004) Factors associated with failure of metronidazole in *Clostridium difficile*-associated disease. J Clin Gastroenterol 38: 414–8. 15100520 10.1097/00004836-200405000-00005

[99] Gasperino J, Garala M, Cohen HW, Kvetan V, Currie B (2010) Investigation of critical care unit utilization and mortality in patients infected with *Clostridium difficile* . J Crit Care 25: 282–6. 19592210 10.1016/j.jcrc.2009.04.002

[100] Pant C, Madonia P, Minocha A, Manas K, Jordan P, et al (2010) Laboratory markers as predictors of mortality in patients with *Clostridium difficile* infection. J Investigat Med 58: 43–5. 10.2310/JIM.0b013e3181bca525 19794314

[101] Venugopal AA, Riederer K, Patel SM, Szpunar S, Jahamy H, et al (2012) Lack of association of outcomes with treatment duration and microbiologic susceptibility data in *Clostridium difficile* infections in a non-NAP1/BI/027 setting. Scand J Infect Dis 44: 243–9. 22077148 10.3109/00365548.2011.631029

[102] de Isusi AM, Gonzalez E, Gayoso Diz P, Gastelu-Iturri J, Barbeito L, et al (2003) *Clostridium difficile*: Experience at a secondary hospital. Med Clin 121 (9): 331–33. 10.1016/s0025-7753(03)73939-6 14499069

[103] Jung KS, Park JJ, Chon YE, Jung ES, Lee HJ, et al (2010) Risk factors for treatment failure and recurrence after metronidazole treatment for *Clostridium difficile*-associated diarrhea. Gut and Liver 4 (3): 332–37. 10.5009/gnl.2010.4.3.332 PMC2956344 20981209

[104] Wilson V, Cheek L, Satta G, Walker-Bone K, Cubbon M, et al (2010) Predictors of death after *Clostridium difficile* infection: a report on 128 strain-typed cases from a teaching hospital in the United Kingdom. Clin Infect Dis 50: e77–81. 20450417 10.1086/653012

