# Correction: Risk Factors for Recurrence, Complications and Mortality in *Clostridium difficile* Infection: A Systematic Review

**DOI:** 10.1371/journal.pone.0107420

**Published:** 2014-08-28

**Authors:** The PLOS ONE Staff

There is an error in Table 1. In the line “Labbé 2008” under “30 Day Mortality”, the values in the columns “N of strains” and “% of strain (n)” should be separated by a backslash. The correct Table 1 can be seen here.

**Table 1:** Association between unfavourable outcomes and strain type in multivariate analyses.

Study	Typing method	Strain type	Period of data collection	N of strains	% of strain (n)	OR/HR/RR (95% CI)
**Recurrence**						
Petrella 2012 [33] / Louie 2013 [36]	REA	BI vs. non-BI	2007- 2009	719	34 (247)^†^	1.57 (1.01- 2.5) / 1.6 (1.03- 2.5)
Stewart 2013 [37]	PCR toxinotyping/ribotyping and tcdC genotyping	Tox A^+^B ^+^ CDT / Tox A^+^B ^+^ CDT + tcdC deletion	NR	69	61 (42)^‡^/ 56 (39)^‡^	3.1 (2.97- 3.3) / 5.3 (3.5- 6.1)
Marsh 2012 [38]	MLVA and tcdC genotyping	tcdC-I genotype (ribotype 027) vs. other	2001- 2009	82	45 (37)	6.9 (1.7- 28.2)
Eyre 2012 [25]	MLST	Ribotype 027 vs. clade 1^a^	2006-2010	1076	28 (300)	1.2 (0.9-1.5)
**Complicated CDI**						
Bauer 2011 [46]	PCR toxinotyping and ribotyping		2008	389		
		Ribotype 018 vs. others			6 (23)	6.2 (1.28- 29.8)
		Ribotype 056 vs. others			2 (6)	13.0 (1.1- 148.3)
		Ribotype 015 vs. others			3 (13)	4.6 (0.98-21.2)
		Ribotype 027 vs. others			5 (19)	2.6 (0.6-10.2)
		Ribotype 014/020 vs. others			16 (61)	0.6 (0.2-2.2)
Søes 2012 [49]	PCR toxinotyping/ribotyping and tcdC genotyping	Tox A^+^B ^+^CDT^+^ vs. A^+^B^+^CDT^-^	2006- 2007	82	26 (21)	6.0 (1.5-23.8)
Walk 2012 [48]	PCR toxinotyping/ribotyping	Ribotype 027/078 vs. others	2010-2011	310	14 (43)	0.8 (0.07-10.0)
Rao 2013 [47]	PCR toxinotyping/ribotyping	Ribotype 027 vs. others	2010—2012	22	32 (7)	2.7 (0.3-25.3)
**30-day mortality**						
Inns 2013 [21]	PCR	Ribotype 027 vs. infrequent ^b^	2009-2011	1426	10 (147)	1.3 (1.02-1.7)
Labbé 2008 [55]	PCR toxinotyping and ribotyping	Ribotype 027 vs. others	2000-01 & 2003-04 (outbreak)	230 / 175	61 (141) / 29 (41)	2.1 (1.0- 4.2) / 7.5 (1.6-35.5)
Walker 2013 [23]	MLST, correlation with ribotypes	Ribotype 027 vs. clade 1^a^	2006-2011	1893	20 (560)	3.4 (2.5-4.7)
Huttunen 2012 [56]	PCR	Ribotype 027 vs. others^c^	2008-2010	780	14 (111)	4.6 (1.4-15)
Goorhuis 2011 [57]	MLVA and STRD	Ribotype 027 vs. others^d^	2005-2007	168	27 (46)	10.5 (1.2-92)
Inns 2013 [21]	PCR	Ribotype 015 vs. infrequent ^b^	2009-2011	1426	8 (111)	0.5 (0.3-0.8)
Goorhuis 2011 [57]	MLVA and STRD	Ribotype 017 vs. others^d^	2005-2007	168	34 (57)	8.9 (1.04-75.8)
Walker 2013 [23]	MLST, correlation with ribotypes	Ribotype 078 vs. clade 1^b^	2006-2011	1893	2 (63)	5.4 (3.1-9.3)
Søes 2012 [49]	PCR toxinotyping/ribotyping and tcdC genotyping	Tox A^+^B ^+^CDT^+^ vs. A^+^B^+^CDT^-^	2006- 2007	82	26 (21)	1.0 (0.2-5.1)

REA = Restriction endonuclease analysis. PCR = Polymerase chain reaction. MLVA = Multiple-Locus Variable number tandem repeat Analysis. STRD = Summed Tandem-Repeat Difference. MLST =  Multilocus Sequence Typing. NR =  not reported;

† Overall % of strain BI in the cohort, the % in the sub-population used for multivariate analyses was not reported.

‡ % of binary toxin gene and *tcd*C mutation respectively, the % of combinations were not reported.

a Comparison of clade 2 with 99% PCR ribotype 027 vs. clade 1, and clade 5 with 100% PCR ribotype 078 vs. clade 1.

b Compared to infrequent ribotypes in the study (other than R01, 02, 05, 015, 016, 023, 027, 064, 078 and 106).

c Hypervirulent strain vs. non-hypervirulent, ribotype 027 was prevailing during the study period.

d Other ribotype : non-027 and non-017.
