# Supplementary material for: Risk Factors for Recurrence, Complications and Mortality in Clostridium difficile Infection: A Systematic Review
Source: PLoS One. 2014 Jun 4;9(6):e98400. doi: 10.1371/journal.pone.0098400 (PMC4045753; doi:10.1371/journal.pone.0098400)
Supplement: Table S1 — Characteristics of included studies addressing risk factors for recurrence [20], [25], [27]–[40], [71], [95], [96]. (PDF) [file pone.0098400.s001.pdf]

Table S1- Characteristics of included studies addressing risk factors for recurrence

Study<br>Year of diagnosis*<br>Country	Design	Diagnostic test	Follow-up period	Time between episodes (days)	Population	Comparison group	Quality variables	Mean/median age ± SD<br>Dispersion	N	% Recurrence (n)	Method	Nv	EPV
Choi 2011 [95]<br>2008-2010<br>Korea	RC	NS toxin essay	NA	<60	Inpatients >15years with CDI, pseudomembranous colitis or diarrhoea	Non recurrent	SI, IS, RS, AB, AU	62.5[¥]<br>15-84	84	13.1 (11)	MLR	11	1
Do 1998 [39]<br>1993-1994<br>Canada	RCC	Toxigenic culture	NA	45	Adult inpatients with ≥ 2 CDI episodes	Patients with 1 CDI episode	SI, PE, IS, AB, AU	77[¥]<br>28-92	59	22 (13)	MLR	4	3.3
Drekonja 2011 [34]<br>2004-2006<br>USA	RC	Culture or NS toxin essay	NA	90	New CDI case in Veterans Affairs	Users/non- users of non-CDI antimicrobials > 30 days after CDI	SI, IS, RS, AB, AU	71±13[§]	246	30 (74)	MLR	8	9.2
Eyre 2012 [25]<br>2006-2010<br>UK	PC	Toxin A and B EIA	≥ 90	≥ 14	Adult inpatients with 1st CDI	Non recurrent	SI, PE, IS, RS	77[¥]<br>IQR=64- 85	1678	22 (393)	Cox hazards model	12	32.7
Fekety 1997 [71]<br>NR<br>USA	RCT	Culture or NS toxin essay	60	< 60	Patients with CDI assigned to placebo in double-blind trial	Patients with a history of recurrent CDI	PE, IS, RS, AB	59.2[§]±21.1	67	51 (34)	MLR	13	1.9
Freedberg 2013 [27]<br>2009- 2012<br>USA	RC	PCR (toxin B)	90	15 to 90	Inpatients with primary CDI	Non recurrent	SI, PE, IS, AB, AU	64[§]±19	894	18.7 (167)	Cox hazards model	10	16.7
Garey 2010 [40]<br>2007-2008<br>USA	PC	Direct CTA	90	< 48h of Abx completion	Inpatients with primary CDI	Non recurrent	RS, AB, AU	61[§]±16	96	24 (23)	MLR	NR	-
Kim 2010 [30]<br>2006-2007<br>Korea	RC	Toxin A and B EIA	90	90	Inpatients with primary CDI	Non recurrent	SI, AB, AU	67.6[§]±13.9	125	21.6 (27)	MLR	4	6.8
Kim 2012 [35]<br>2004-2008<br>Korea	RCC	Toxin A and B EIA	NA	30	Patients with primary CDI	Non recurrent	SI, PE, IS, RS, AB, AU	66.8[§]±2.7	42	14.1 (28)	Propensity score MLR	7	3.1
Kyne 2001[32]<br>1998<br>USA	PC	Toxin A EIA or direct CTA	60	> 48h	Inpatients with CDI	Patients with one CDI episode	IS, AU	70±20[§]<br>34-95	63	35 (22)	MLR	10	2.2
Lavergne 2013 [29]<br>2009-2010<br>Canada	PC	2-step approach: glutamate dehydrogenase (GDH) EIA, and direct CTA	60	< 60 of treatment completion	Adult inpatients with primary CDI	Non recurrent	SI, PE, AB, AU	77[¥]<br>65-84	121	33.1 (40)	Cox hazards model	4	10

Study<br>Year of diagnosis*<br>Country	Design	Diagnostic test	Follow-up period	Time between episodes (days)	Population	Comparison group	Quality variables	Mean/median age ± SD Dispersion	N	% Recurrence (n)	Method	Nv	EPV
Linsky 2010 [20]<br>2004-2008<br>USA	RC	Toxin A and B EIA	NA	15-90	Patients from VA with CDI exposed to PPIs	Patients not exposed to PPIs	SI, IS, AB, AU	74[¥]<br>63-82	1166	21.5 (251)	Cox hazards model	10	25.1
Louie 2013 [36]<br>2006- 2009<br>Multicentre	RCT	Toxin A or B EIA	40	28	CDI patients randomly receiving oral vancomycin or fidaxomicin	Non recurrent	PE, AB	NR	567	NR	MLR	4	-
Marsh 2012 [38]<br>2001- 2009<br>USA	RC	Direct CTA	NA	14	Patients with recurrent CDI	Patients with reinfection	PE, IS, AB, AU	64[¥]	82	62.2 (51)	MLR	13	4
McFarland 1999 [28]<br>1993-1996<br>USA	RCT	Culture, toxin A EIA or direct cytotoxin assay	60	60	Patients with recurrent CDI treated with Abx and placebo in a clinical trial	Non recurrent	PE, AB	62.1±18.5[§]	103	41.7 (43)	MLR	15	2.9
Pepin 2005 [31]<br>1991-2004<br>Canada	RC	Direct CTA	NA	60	Inpatients with CDI	Non recurrent	SI, PE, IS, RS, AB	All ages	2042	11.9 (243)	Cox hazards model	9	27
Petrella 2012 [33]<br>2006- 2009<br>Multicentre	Same as Louie 2013 [36]						PE, AB	NR	999	18.9 (150/794)	MLR	9	16.7
Shakov 2011[96]<br>2003-2008<br>USA	RC	NS toxin A and B assay	NA	< 180	Inpatients with CDI	Patients without readmission for CDI	AB, AU	74[¥]<br>59.8- 82.0	247	30.7 (76)	MLR	7	10.9
Stewart 2013[37]<br>NR<br>USA	RC	PCR on positive culture	NR	21	Inpatients and outpatients with *C. difficile* positive stool	Non recurrent	RS, AU	64± 13[§]	69	41 (28)	MLR	NR	-

Nv= number of variables in the final model. EPV=events per variable. MLR=multivariate logistic regression. NA=not applicable. NR=not reported.
*Year of diagnosis=year(s) of cases diagnosis. § Mean age; ¥ Median age.
Design= RC=retrospective cohort; PC=prospective cohort; RCC=retrospective case-control; PCC=prospective case-control.
NS=Not specified. EIA=Enzyme immunoassay. CTA=cytotoxin assay. PCR=Polymerase chain reaction;
Quality variables: SI= site of acquisition of the infection (nosocomial vs. community-acquired), PE= previous episode(s) of CDI, IS= immunosuppression, RS= recent surgeries and procedures, AB= recent antibiotherapy, AU= use of anti-ulcer medication.
