# Supplementary material for: Risk Factors for Recurrence, Complications and Mortality in Clostridium difficile Infection: A Systematic Review
Source: PLoS One. 2014 Jun 4;9(6):e98400. doi: 10.1371/journal.pone.0098400 (PMC4045753; doi:10.1371/journal.pone.0098400)
Supplement: Table S2 — Characteristics of included studies addressing risk factors for complicated CDI and treatment failure [41], [42], [44], [45], [47], [48], [50], [85]–[88], [97], [98]. (PDF) [file pone.0098400.s002.pdf]

Table S2- Characteristics of included studies addressing risk factors for complicated CDI and treatment failure

Study<br>Year of diagnosis*<br>Country	Main outcome definition	Design<br>Follow-up	Diagnostic test	Population	Comparison group	Quality variables	Mean/ median age ± SD<br>Dispersion	N	% Outcome (n)	Method	Nv	EPV
Andrews 2003 [45]<br>1995-1999<br>Canada	Hospital stay ≥ 14 days, colectomy, ICU admission, death	RC	Toxin A EIA	Primary CDI discharge (ICD-10 008.45)	Patients with mild CDI	SI, IS, RS, AB, AU	63.4[§]	153	28.8 (44)	MLR	3	14.7
Greenstein 2008 [88]<br>1994-2006<br>USA	**Fulminant colitis**: pathologically confirmed pseudomembranous colitis after emergent colectomy	RCC	Toxin A and B EIA	Adult inpatients with fulminant CDI	Patient with nosocomial & community-acquired CDI	SI, PE, IS, AB, AU	70[§]	105<br>(35 vs. 70)	-	MLR	9	3.8
Gujja 2009 [97]<br>2003-2008<br>USA	**Complication**: colon resection or death	RC	Toxin A and B EIA	Primary CDI discharge (ICD-9 008.45)	Patients without complication	IS	68.8[§]±12.9	200	16 (32)	MLR	3	7.3
Hardt 2008[86]<br>2003-2006<br>Germany	**Severity:** profuse diarrhoea and positive shock index	RC	Direct CTA	Adult inpatients	Patients with non- severe CDI	SI, IS, RS, AB, AU	76[§]	124	21.7 (27)	MLR	9; 2 in reduced model	3
Henrich 2009 [41]<br>2005-2006<br>USA	**Severity:** 30-day death or ≥1 ICU admissions, colectomy, intestinal perforation	RC	Direct CTA and toxin A and B EIA	Inpatients non-ambulatory nor emergency	Patients with non-severe CDI	IS, RS, AU	All ages<br>≥ 18years	336	12.2 (41)	MLR	9	4.5
Kyne 1999 [87]<br>1995<br>Ireland	**Progression to severe disease**: associated morbidity, malnutrition, faecal incontinence, toxic megacolon or death	PC<br>Until disch-arge or death	Direct CTA	Adult inpatients	Patients with mild /moderate CDI	IS, RS, AB, AU	74[¥]<br>17-91	73	39.7 (29)	MLR	11/ 3 in reduced model	2.6
Manek 2011 [44]<br>2007-2008<br>Canada	**Severe complications:** hypokalaemia, toxic megacolon, bowel perforation, lower gastrointestinal bleeding, ICU transfer or death	RC	Toxin A and B EIA	Inpatients with CDI	Inpatients with CDI without complications	SI, PE, RS	71[§]±16	365	26.6 (97)	MLR	13	7.5
Pepin 2004 [42]<br>1991-2003<br>Canada	**Complication:** toxic megacolon, perforation, colectomy or shock requiring vasopressor therapy or all-cause 30-day death	RC	Direct CTA	Inpatients with CDI	Patients without complications	SI, PE, IS, RS, AB	All ages	1721	10.6 (182)	Uncon-ditional MLR	8	22.7

Study<br>Year of diagnosis*<br>Country	Main outcome definition	Design<br>Follow-up	Diagnostic test	Population	Comparison group	Quality variables	Mean/ median age ± SD Dispersion	N	% Outcome (n)	Method	Nv	EPV
Rao 2013 [47]<br>2010- 2012<br>USA	**Severity:** WBC > 15x10^3/mm^3, temperature > 38°C, or acute organ dysfunction (clinical score), and/or no initial response to therapy, and ICU stay, colectomy, 30-day attributable death	PC	GDH + Toxin A and B EIA/ PCR when GDH+/toxin-	Inpatients with CDI	-	SI, IS, AB	58[¥]<br>20- 88	69	30.4 (21 clinical score)/ 39.1 (27 expanded score)	MLR	7	4 (expand-ed score)
Walk 2012 [48]<br>2010- 2011<br>USA	**Severity:** ICU admission, surgery or 30-day death	RC	PCR on positive culture	Suspected CDI inpatients and ambulatory outpatients	-	SI	57[¥]<br>0.5- 93	310	11 (34)	MLR	11	3.1
Wenisch 2012 [85]<br>2009-2010<br>Austria	**Severe CDI**: ICU admission, surgery for CDI, death directly or contributively due to CDI	RC	Toxin A and B EIA and PCR	Hospital-acquired CDI in medicine wards	Patients with non-severe CDI	SI	74.4[§]<br>37-92	133	18.1 (24)	MLR	4	6
Treatment failure considered alone												
Fernandez 2004 [98]<br>2000-2001<br>USA	**Failure of MTZ**: persistence of fever or abdominal pain or ≥3 bowel movements/ day after 5 full days of treatment	RC	Toxin A EIA	Inpatients with CDI who received MTZ as initial therapy for ≥5 days	Patients who improved with MTZ	RS, AB	66[§]<br>23-96	99	38.4 (38)	MLR	7	5.4
Hu 2008 [50]<br>2004-2006<br>USA	**Failure of MTZ**: persistence of diarrhoea for ≥10 days or clinical decision to start vancomycin before 10-days endpoint	PC<br>60 days	NS toxin assay	Adult inpatients with CDI	Patients with CDI before NAP-1 strain (1998)	SI, PE, IS, AB, AU	70[¥]<br>22-94	89	31.5 (28)	MLR	4	7

Nv= number of variables in the final model. EPV= events per variable. NR= not reported. MTZ= metronidazole.
*Year of diagnosis= year(s) of cases diagnosis.
§ Mean age; ¥ Median age.
Design: RC= retrospective cohort; PC=prospective cohort; RCC=retrospective case-control; PCC=prospective case-control.
Diagnostic test: NS= Not specified, EIA= Enzyme immunoassay, CTA= cytotoxin assay, PCR= Polymerase chain reaction;
Quality variables: SI= site of acquisition of the infection (nosocomial vs. community-acquired), PE= previous episode(s) of CDI, IS= immunosuppression, RS= recent surgeries and procedure, AB= recent antibiotherapy, AU= use of anti-ulcer medication.
