# Supplementary material for: Risk Factors for Recurrence, Complications and Mortality in Clostridium difficile Infection: A Systematic Review
Source: PLoS One. 2014 Jun 4;9(6):e98400. doi: 10.1371/journal.pone.0098400 (PMC4045753; doi:10.1371/journal.pone.0098400)
Supplement: Table S3 — Characteristics of included studies addressing risk factors for mortality considered alone [21]–[24], [53], [55]–[57], [59]–[63], [72]–[74], [89], [90], [92], [94], [99]–[101]. (PDF) [file pone.0098400.s003.pdf]

Table S3- Characteristics of included studies addressing risk factors for mortality considered alone

Study<br>Year of diagnosis*<br>Country	Definition of mortality	Design<br>Follow-up	Diagnostic test	Population	Comparison group	Quality variables	Mean/ median age ±SD Dispersion	N	% Mortality (n)	Method	Nv	EPV
Bishara 2008 [92]<br>1999-2000<br>Israel	Short and long-term	PC<br>28 days	Toxin A and B EIA	Inpatients with diarrhoea who received antibiotics ≤40 days prior to diarrhoea	Inpatients with negative *C.difficile* toxins	PE, IS, RS, AB, AU	71.5¥<br>18-94	217	15.4 (8)	Cox hazards model	4	2
Das 2010 [94]<br>2004-2008<br>USA	All-cause 30-day	RC	Toxin A and B EIA	Inpatients with CDI	Patients who did/ not receive glucocorticoids ≤15 days of CDI	IS	62.2§±19.8	2024	11 (223)	Cox hazards model	6	37.2
Gasperino 2010 [99]<br>2005<br>USA	All-cause 30-day	RC	Toxin A and B EIA	Inpatients with CDI	Inpatients with negative *C.difficile* toxins	SI, PE, AB, AU	68§ ±17	216	16 (17)	MLR	9	1.8
Goorhuis 2011[57]<br>2005-2007<br>The Netherlands	Overall, attributable and contributable 30-day	RCC	Toxin A and B EIA	Inpatients with CDI	Inpatients with non-CDI diarrhoea	IS, RS, AB	NR	245	17.2 (16), 4.8 (8) and 8.4 (14)	MLR	6	2.7
Huttunen 2012 [56]<br>2008-2010<br>Finland	All-cause 30-day	RC	PCR on positive culture	Patients with cultures positive for *C. difficile*	Survivors	None	77¥<br>17-100	780	10.3 (80)	MLR	4	20
Inns 2013 [21]<br>2009-2011<br>England	All-cause 30-day	RC<br>92-821 days	NS toxin assay	Patients with toxin-positive CDI	Survivors	SI	79¥<br>71- 86	1426	25.7 (366)	Poisson regression	7	52.2
Jansen 2010 [89]<br>2007<br>Germany	CDI-related 30-day	RC	Toxigenic culture	Patients with severe CDI	Survivors	SI, PE, AB, IS, RS	NR	48	19 (9)	MLR	6	1.5
Keneally 2007 [72]<br>2004-2005<br>USA	All-cause 30-day	RC	Toxin A and B EIA	Adult inpatients in an ICU with CDI	Survivors	SI, PE, IS, RS, AB	63.9 ¥<br>17-96	278	36.7 (102)	MLR	6	17
Khan 2012 [90]<br>2006- 2009<br>Qatar	All-cause 30-day	RC	Toxin A and B EIA	Inpatient with CDI	Survivors	SI, IS, RS, AB, IPP	50.9§±21.2<br>15- 99	123	30.9 (38)	MLR	5	7.6
Labbé 2008 [55]<br>2000-01/<br>2003-04<br>Canada	All-cause 30-day	PC	Toxigenic culture	Adults in and outpatients	Survivors	SI, PE, AB	≥20	230	23.9 (55)	MLR	4	13.7
Lamontagne 2007 [73]<br>2003-2005<br>Canada	All-cause 30-day of ICU admission	RC	Direct cytotoxin assay	Patients with CDI requiring admission to ICU	Survivors	SI, PE, IS, AB	75¥<br>39-93	165	52.7 (87)	MLR	5	17.4
Marra 2007 [53]<br>2002-2005<br>USA	14-day	RC	Direct CTA	ICU patients	Survivors	SI, RS, AB	55.5¥<br>43-64	58	27.6 (16)	MLR	7	2.3
Pant 2010 [100]<br>NR (10 years)<br>USA	All-cause 30-day	RC	Toxin A and B EIA	Inpatients with CDI	Survivors	PE	NR	184	13.6 (25)	MLR	7	3.6

Study<br>Year of diagnosis*<br>Country	Definition of mortality	Design Follow -up	Diagnostic test	Population	Comparison group	Quality variables	Mean/ median age ±SD Dispersion	N	% Mortality (n)	Method	Nv	EPV
Sailhamer 2009 [74]<br>1996-2007<br>USA	Inpatient	RC	Toxin A and B EIA	Inpatients with fulminant colitis	Survivors	PE, IS, RS, AB	68.3§± 15.6	199	34.7 (69)	MLR	15	4.6
Stewart 2011[22]<br>2007<br>USA	Excess attributable mortality	RC	NS	Inpatients with CDI	Inpatients without CDI	None	70§	41207	9.4 (3873)	MLR with propensity scores	9	430
Venugopal 2012 [101]<br>2005-2006<br>USA	All-cause 30-day	PC	Toxin A and B EIA	Inpatients with CDI	Survivors	SI, PE, IS, RS, AB	67.9§ ± 16.8	118	24.6 (29)	MLR	7	4.1
Walker 2013 [23]<br>2006- 2011<br>UK	14-day attributable mortality	RC	EIA	Adult patients with positive EIA test for CDI	EIA-negative tests	SI, PE	78¥<br>67- 85	2745 vs. 27550	7.7 (1847)	Cox hazards model	18	103
Mortality in patients undergoing surgery												
Byrn 2008 [59]<br>1994-2005<br>USA	In-hospital	RC	Toxin A and B EIA	Patients undergoing colectomy for fulminant colitis	Survivors	PE, IS, RS	68§	73	34.2 (25)	MLR	4	6.2
Dias-Perera 2007 [60]<br>1997-2005<br>USA	30-days of surgery	RC	NS	Patients undergoing colectomy for fulminant colitis	Survivors	RS	69.7§	35	45.7 (16)	MLR	3	5.3
Halabi 2013 [24]<br>2001- 2010<br>USA	In-hospital	RC	NS	Inpatients who underwent colectomy for CDI	Survivors	RS	NR	19374	30.7 (5949)	MLR	17	350
Markelov 2011 [61]<br>2000-2009<br>USA	In-hospital	RC	NS toxin assay or NS PCR	Patients with surgical treatment for CDI colitis	Survivors	RS, AB	74§ ±8.2	13	38.5 (5)	MLR	8	0.6
Pepin 2009 [62]<br>1994-2007<br>Canada	30-days of surgery	RC	Direct CTA	Inpatients with CDI who underwent a colectomy or hemicolectomy	Survivors	SI, PE, IS, RS	71.5¥<br>20-88	130	37 (48)	MLR	7	6.8
Seder 2009 [63]<br>2000-2007<br>USA	Inpatients	RC	NS	Adults with surgical management for CDI	Survivors	PE, IS, AU	71§	69	42 (29)	MLR	4	7.2

Nv= number of variables in the final model. EPV= events per variable. MLR=multivariate logistic regression. NR= not reported;
*Year of diagnosis= year(s) of cases diagnosis;
§ Mean age. ¥ Median age;
Design: RC= retrospective cohort; PC=prospective cohort; RCC=retrospective case-control; PCC=prospective case-control.
Diagnostic test: NS= Not specified, EIA= Enzyme immunoassay, CTA= cytotoxin assay, PCR= Polymerase chain reaction.
Quality variables: SI= site of acquisition of the infection (nosocomial vs. community-acquired), PE= previous episode(s) of CDI, IS= immunosuppression, RS= recent surgeries and procedures, AB= recent antibiotherapy, AU= use of anti-ulcer medication.
