# Supplementary material for: Risk Factors for Recurrence, Complications and Mortality in Clostridium difficile Infection: A Systematic Review
Source: PLoS One. 2014 Jun 4;9(6):e98400. doi: 10.1371/journal.pone.0098400 (PMC4045753; doi:10.1371/journal.pone.0098400)
Supplement: Table S4 — Characteristics of included studies addressing risk factors for multiple outcomes [26], [43], [46], [49], [51], [52], [54], [58], [91], [93], [102]–[104]. (PDF) [file pone.0098400.s004.pdf]

Table S4- Characteristics of included studies addressing risk factors for multiple outcomes

Study<br>Year of diagnosis*<br>Country	Main outcome(s)' definition	Design<br>Follow-up (days)	Diagnostic test	Population	Comparison group	Quality variables	Mean/ median age ±SD Dispersion	N	% Rec[‡] (n)	% Com[†] (n)	% Death (n)	Method	Nv	EPV
Bauer 2011[46]<br>2008<br>34 European countries	**Recurrence**<br>**Complication:** ICU admission or colectomy, or death	PC<br>90	Toxin A and B EIA, direct CTA, PCR	Out and inpatients with CDI	Non-recurrent and cured patients	SI, PE, AB, IS	71[¥]<br>IQR=56-81	509	18<br>(86)	7.4<br>(34)	22 (101)	2-levels MLR	13[‡]<br>/15[†]	6.6[‡] /<br>2.9[†]
Cadena 2010 [54]<br>2003-2005<br>USA	**Recurrence:** recurrent symptoms of CDI after completion of therapy and initial complete resolution;<br>**90-day death**	RC<br>>90	Toxin A and B EIA	Inpatients in veterans' health care system	Non-recurrent CDI	SI, PE, IS, RS, AB, AU	All ages	129	29<br>(38)	-	29 (38)	MLR	3	12.7
Cloud 2009 [43]<br>2004-2006<br>USA	**Severe CDI**: death or CDI as contributor, ICU admission or with CDI as contributing factor, toxic megacolon or colectomy;<br>**60-day inhospital death**<br>**Treatment failure:** change from MTZ to or addition of vancomycin	PC<br>60	Toxin A EIA	Inpatients with CDI	Cured patients Survivors	SI, AB, AU	67.3[§]	272	-	22.1<br>(60)<br>21<br>(53) [£]	4 (11)	MLR	5	12
Cober 2009 [51]<br>2006<br>USA	**90-day death**<br>**Treatment failure:** lack of improvement after 5 days or change in treatment regimen	RC	Toxin A or B EIA	80 and older inpatients	Survivors	RS, AB, AU	84 ± 4.1[§]<br>80-94	70	17<br>(12)	25.7<br>(18)[£]	17.1 (12)	MLR	9	1.3
de Isusi 2003 [102]<br>1999-2001<br>Spain	**Failure or recurrence**: absence of clinical resolution or reappearance of clinical symptoms ≥7 days;<br>**Death:** Inhospital all-cause	RC	Toxin A EIA	Inpatients CDI	Cured patients Survivors	SI, PE, IS, RS, AB, AU	71.7[§]<br>20-99	113	18<br>(20)	20.7<br>(23)[£]	30.1 (34)<br>19.5 (22) CDI related	MLR	7	2.6

Khanna 2012 [52] 1991- 2005 USA	**Recurrence:** CDI within 8 weeks after symptom resolution; **Severe-complicated:** hypotension, sepsis, ileus, toxic megacolon, perforation, need for ICU admission, surgery or 30-day death; **Treatment failure:** persistent symptoms leading to change initial treatment within 14 days due to nonresponse or intolerable adverse effects	RC	Direct CTA	All cases of CDI	None	SI, PE, AB, AU	67.6¥ 10d- 102yrs	385	30.1 (116)	7 (27)/ 20.2 (75) £	-	MLR	5	23.2‡/ 5.4†
Kim 2013[91] 2005- 2010 South Korea	**Response failure to treatment**: no improvement in diarrhoea by day 10 **All cause 30-day mortality**	RC 30	Toxin A EIA (2005-2008); Toxin A and B EIA (2008-2010)	Patients with CDI	Response/ survivors	AB	64.4§ ±14.1	536	-	4.9£ (26)	9 (48)	MLR	4£/6	6.5£/8
Jung 2010 [103] 1998-2008 South Korea	**Recurrence**: reappearance of symptoms or positive toxin assay within 90 days of treatment response **Treatment failure:** persistence or incomplete resolution of symptoms, or positive toxin assay, >10 days of treatment	RC	Toxin A EIA	Adult inpatients	Non-recurrent and cured patients	IS, AB, AU	62.5±13.8§ 23-88	117	11 (13.4)	12.6 (14)£	-	MLR	3£	4.3
Morrison 2011 [58] 2004-2008 USA	**Complications:** CDI-associated mortality, surgery and toxic megacolon	RC	NS toxin assay	Active-duty military, spouses, children and retirees	NR	IS, RS, AB, AU	53¥ 1-99	485	-	9.7 (47)	4.7 (23)	MLR	4†/2	11.7†/ 11.5
Pepin 2006 [26] 1991-2005 Canada	**Recurrence** **Complications during 1st recurrence :** toxic megacolon, perforation, colectomy, shock requiring vasopressors or 30-day all-cause death	RC 60	Direct CTA	Patients who experienced at least 1 recurrence	Patients without a 2nd recurrence	SI, IS, AB	All ages	463	63.6 (98)	11 (51)	9.3 (43)	Cox hazards model	3‡/6†	32.7‡/ 8.5†

Søes 2012 [49]<br>2006- 2007<br>Denmark	**Clinical severity score (≥1.5 pts)**: level of diarrhoea, systemic symptoms, result of sigmoïdoscopy<br>**Mortality**: 30- day	PC<br>30	PCR on positive culture	Inpatients positive for *C. difficile* stool culture	None	SI, IS, RS, AB	69¥	82	-	47.6<br>(39)	11 (9)	MLR/<br>Cox hazards model	6	6.5/<br>1.5
Solomon 2013 [93]<br>2008- 2009<br>Ireland	**All-cause 30-day mortality**<br>**Mortality directly attributable to CDI**<br>**Recurrence** 60 days after resolution of primary diarrhoeal episode	PC	Toxin A and B EIA	Consecutive CDI cases	Survivors	None	74.5§<br>35-97	86	18.6<br>(16)	-	16.3 (14)	MLR	5	2.8
Wilson 2010 [104]<br>2007-2008<br>UK	**Mortality**: 30-day all-cause and CDI-related<br>**Early treatment failure:** change of 1st choice, dose, or route of administration	PC<br>30	Toxin A and B EIA	New CDI cases	Survivors B1/027, DH/106 and other strains	SI, IS	NR	128	19.6<br>(22)	50<br>(57)£	35.9 (46) overall<br>17.2 (22) CDI related**	MLR	6	3.7**

Nv= number of variables in the final model. EPV= events per variable. MLR=multivariate logistic regression. NR= not reported.
*Year of diagnosis= year(s) of cases diagnosis.
‡ Rate or % of recurrence. † Rate or % of complicated CDI. £ Treatment failure. ** Death in treated patients.
§ Mean age. ¥ Median age.
Design: RC= retrospective cohort; PC=prospective cohort; RCC=retrospective case-control; PCC=prospective case-control.
Diagnostic test: NS= Not specified, EIA= Enzyme immunoassay, CTA= cytotoxin assay, PCR= Polymerase chain reaction.
Quality variables: SI= site of acquisition of the infection (nosocomial vs. community-acquired), PE= previous episode(s) of CDI, IS= immunosuppression, RS= recent surgeries and procedures, AB= recent antibiotherapy, AU= use of anti-ulcer medication.
