# Supplementary material for: Risk Factors for Recurrence, Complications and Mortality in Clostridium difficile Infection: A Systematic Review
Source: PLoS One. 2014 Jun 4;9(6):e98400. doi: 10.1371/journal.pone.0098400 (PMC4045753; doi:10.1371/journal.pone.0098400)
Supplement: Text S1 — Electronic search: databases and keywords. (PDF) [file pone.0098400.s006.pdf]

Text S1- Electronic search

Databases used for the electronic search

- MEDLINE: Ovid MEDLINE®, In-Process & Other non-indexed citations since 1950 and PubMed
- Cochrane Library: EBM Reviews - Cochrane Central Register of Controlled Trials 4th Quarter 2013, EBM Reviews - Cochrane Database of Systematic Reviews 2005 to October 2013
- Embase: 1980-2011
- Web of Sciences: SCI-EXPANDED 1979-2011

Keywords

(*Clostridium difficile* OR *Clostridium difficile*-associated diarrhoea OR *Clostridium difficile*-associated disease) AND (diarrhoea OR diarrhoea OR colitis OR pseudomembranous OR enterocolitis OR enteritis OR antibiotic-associated disease) AND (sensitivity OR specificity OR predict$ OR index OR score OR model OR factor OR grad$ OR decision rule OR decision technique OR prognosis OR risk index OR risk score OR risk model OR risk scale).
